# Supplementary material for: Protocol for a realist evaluation of Recovery College dementia courses: understanding coproduction through ethnography
Source: BMJ Open. 2023 Dec 7;13(12):e078248. doi: 10.1136/bmjopen-2023-078248 (PMC10711820; doi:10.1136/bmjopen-2023-078248)
Supplement: Supplementary data [file bmjopen-2023-078248supp001.pdf]

Supplementary file 1

Recovery Colleges

The first UK Recovery College was set up in 2009, growing to 85 by 2017 (Anfossi, 2017). The model has been replicated internationally (Perkins et al., 2018) with Recovery Colleges now in existence or developing in 22 countries (King & Meddings, 2019). Recovery College courses offer access to distinctive peer support both from co-producing courses and/or attending them (Sommer, Gill & Stein-Parbury, 2018). A national strategy to promote dementia peer support through developing learning networks also aims to empower people living with dementia to make choices in planning for the future (Department of Health, 2009). A 'recovery-focused', peer-support, adult learning approach is adopted and embedded within a strategic care delivery approach in mental health Trusts, to improve care outcomes beyond a narrow focus on symptom reduction, to help people rebuild meaningful, satisfying lives, despite limitations caused by mental health difficulties (Perkins et al., 2018). Recovery Colleges create adult learning environments which moderate power dynamics between service users and staff, to reduce stigma and increase attendees' sense of hope and empowerment (Meddings et al., 2014; Sommer, Gill & Stein-Parbury, 2018; Zabel et al., 2016). Attendees report developing novel coping strategies, improving self-worth, wellbeing and quality of life (Meddings et al., 2014; Rinaldi, Marland & Wybourn, 2011; Secker & Wilson, 2014; Zucchelli & Skinner, 2013).

A typical adult mental health Recovery College offers courses on mental health and recovery, designed to increase attendees' knowledge, skills and confidence in self-management of their own mental health and wellbeing. Of note, what 'wellbeing' means for people with dementia may be very different, but has been defined as emotional (positive states), social (connections and belonging) and psychological (positive sense of self, going beyond and agency and purpose) with an overarching theme of 'valuing life', which differs to studies evaluating life satisfaction (Clarke et al., 2020). Courses range from one-off sessions to several sessions spread over a set number of weeks. All courses are co-produced and co-delivered (i.e. co-taught) by peer tutors - that is, people with lived/expert experience - and mental health staff, who also enrol on courses as attendees. Peer tutors prepare for their role through having training to teach and support, and receive supervision to ensure any sensitive issues can be supported effectively.
A theory of change model for Recovery Colleges has been co-developed within adult mental health contexts that identifies four mechanisms of change (Toney et al., 2018) empowering environment - opportunities for choices; shifting balance of power; enabling different relationships and connecting with peers; and facilitating personal growth through shared learning and strength-building. Outcomes were changes in the attendee including improved wellbeing, reinforced by life changes they could observe. This model is highly applicable to enabling desired outcomes for post-diagnostic support in dementia.

A 'recovery-focused', peer-support, adult learning approach is adopted and embedded within a strategic care delivery approach in mental health Trusts, to improve care outcomes beyond a narrow focus on symptom reduction, to help people rebuild meaningful, satisfying lives, despite limitations caused by mental health difficulties (Perkins et al., 2018). This approach is already flourishing in adult mental health services, encouraged since 2011 by the Department of Health commissioned 'Implementing Recovery through Organisational Change' (ImROC) collaborative https://imroc.org/. Working with mental health Trusts, ImROC founded Recovery Colleges which have rapidly become a core part of recovery-focused mental health services. As of 2017, there were 85 Recovery Colleges in the UK (77 in England, 2 in Scotland, 5 in Northern Ireland and 1 in Wales; Anfossi, 2017).

Recovery Colleges further have five key linked conceptual processes - the **CHIME** recovery framework – robustly developed to underpin the term 'recovery' in this context: **connecting** with others, inspiring **hope**, maintaining a positive **identity**, finding **meaning** in life outside of symptoms and **empowering** control over life and a focus on strengths (Leamy et al., 2011).

The CHIME Recovery framework, as operationalised through Recovery Colleges, has clearly relevant links with the NICE-recommended person-centred care framework for dementia (Leamy et al., 2011; Brooker, 2007), the Royal College of Psychiatrists Memory Services National Accreditation Programme (MSNAP; Copland et al., 2018) and the National Dementia Strategy objective to develop peer support and learning networks (Department of Health, 2009). Key domains for person-centred care are **valuing** people living with dementia and those (both informal family and friends and health and social care staff) who care for them; providing care that is **individualised**; understanding and acting from the **perspectives** of people living with dementia (which can reinforce connections, meanings and identities); and creating positive **social-psychological** environments (which can build hope and empowerment; Brooker, 2007). For dementia care to be person-centred, all these elements are needed and need to work together. Both frameworks require mental health services to continuously improve, and to evidence progress towards more meaningful care within each of these domains (Brooker, 2007; Leamy ey al., 2011,) yet person-centred principles are still poorly and inconsistently translated into practice. Applying these frameworks in memory post-diagnostic support services is important for keeping a whole person focus so as to value the identity, perspectives, strengths and needs of people living with dementia, and so to enable staff to share with patients more effectively care planning alongside understandings of personal recovery in adjusting to dementia.

Five recovery processes giving the acronym CHIME

Connectedness with others;
inspiring **Hope** and optimism about the future;
maintaining a positive **Identity**;
finding **Meaning** in life outside of symptoms;
and **Empowerment** with control over life and a focus on strengths. (Leamy et al., 2011)

References

Anfossi A. The current state of Recovery Colleges in the UK : final report. *ImROC.* 2017.
Brooker D. *Person-centred Dementia Care: Making services better.* London: Jessica Kingsley; 2007.
Clarke C, Woods B, Moniz-Cook E, Mountain G, Øksnebjerg L, Chattat R, Diaz A, Gove D, Vernooij-Dassen M, Wolverson E. Measuring the well-being of people with dementia: a conceptual scoping review. *Health and quality of life outcomes.* 2020;18(1), 1–14
Copland E, Hodge S, Clary L, Cartwright V. *Memory Services National Accreditation Programme (MSNAP) Standards: Standards for Memory Services*. London; 2018. Available from: https://www.rcpsych.ac.uk/docs /default-source/improving-care/ccqi/quality-networks/memory-clinics-msnap/msnap-standards-6th-edition-2018.pdf [Accessed 3rd March 2021].
Department of Health. *Living well with dementia: A National Dementia Strategy.* 2009. Available from: https://assets.publishing.service.gov.uk/government/uploads/system/uploads/attachment_data/file /168220/dh_094051.pdf [Accessed 10th December 2019].
King T, Meddings S. Survey identifying commonality across international Recovery Colleges. *Ment Heal Soc Incl.* 2019;23(3):121–8.

Leamy M, Bird V, Le Boutillier C, Williams J, Slade M. Conceptual framework for personal recovery in mental health: Systematic review and narrative synthesis. *Br J Psychiatry*. 2011;199(6):445–52.
Meddings S, Byrne D, Barnicoat S, Campbell E, Locks L. Co-Delivered and Co-Produced: Creating a Recovery College in Partnership. Jce . Vol 9 (1); pp16-25. *J Ment Heal Training, Educ Pract.* 2014;9(1):16–25.
Meddings S, McGregor J, Roeg W, Shepherd G. Recovery Colleges: Quality and outcomes. *Ment Heal Soc Incl*. 2015;19(4):212–21.
Perkins R, Meddings S, Williams S, Repper J. Recovery Colleges 10 Years On. *ImROC.* 2018
Rinaldi M, Marland M, Wybourn S. *Annual Report 2011 – 2012 South West London Recovery College*. 2012. Available from: http://rfact.org.au/wp-content/uploads/2015/05/SW-London-Recovery-College-evaluation-2011_12-v1-0.pdf [Accessed 12th February 2020].
Secker J, Wilson C. *Evaluation of the mid Essex Recovery College, October – December 2013. 2014.* Anglia Ruskin University, 2014.
Sommer J, Gill K, Stein-Parbury J. Walking side-by-side: Recovery Colleges revolutionising mental health care. *Ment Heal Soc Incl*. 2018;22(1):18–26.
Toney R, Elton D, Munday E, Hamill K, Crowther A, Meddings S, et al. Mechanisms of action and outcomes for students in recovery colleges. *Psychiatr Serv.* 2018;69(12):1222–9.
Zabel E, Donegan G, Lawrence K, French P. Exploring the impact of the recovery academy: a qualitative study of Recovery College experiences. *J Ment Heal Training, Educ Pract*. 2016;11(3):162–71.
Zucchelli FA, Skinner S. Central and North West London NHS Foundation Trust's (CNWL) Recovery College: the story so far. *Ment Heal Soc Incl*. 2013;25
