# Supplementary material for: Protocol for a realist evaluation of Recovery College dementia courses: understanding coproduction through ethnography
Source: BMJ Open. 2023 Dec 7;13(12):e078248. doi: 10.1136/bmjopen-2023-078248 (PMC10711820; doi:10.1136/bmjopen-2023-078248)

Supplementary file 2 Valid Informed Consent Processes

Process of obtaining consent from each co-production group The co-production group at each site will be contacted first and we aim to be as flexible and pragmatic as possible in recruiting and seeking informed consent from individuals in the group so we can begin observing co-production activities and documents as soon as possible prior to the researcher-observed course starting.

- Two case study researchers arrange an informal pre-course meeting with the co-production group to seek permission to contact for individual consent meeting/s
- Facilitate introductions, give information about the study, answer any questions, discuss and plan to join further meetings as ethnographic observers of the co-production process
- Gain understanding of practicalities around course publicity, set up and co-facilitation
- Discuss what types of documents and materials might be available for researchers to review

Process of obtaining consent from attendees Similarly we aim to be as flexible and pragmatic as possible in recruiting and seeking informed consent from all dementia course attendees. When people enrol on the Recovery College dementia course, they will be advised of the planned research and invited to find out more about the study. If at this point people do not wish to be involved in the research, the Lead Professional will coordinate their individual booking to alternative courses/dates.

If a person agrees in principle, the first line of obtaining consent will be for attendees who inform the case site Lead Professional they are planning to attend the course. The Lead Professional/site staff will provide the attendee with a participant information sheet. If agreed with the potential attendee, a ‘permission to contact’ form will be completed by the Lead Professional/site staff and sent securely via email to the study team via the DiSCOVERY study email address. The study team will attempt to obtain informed consent from all attendees, including healthcare professionals, people with dementia and their family supporters via remote methods prior to the day of data collection. If remote methods of informed consent are not possible, attendees will be offered the opportunity to speak to the study team in person prior to the course and provide informed consent on the day. However it is possible that people will attend the course without providing prior consent for taking part in the study. In those cases, attendees will be provided study information and offered the opportunity to take part in the research study immediately prior to the data collection commencing in accordance with HRA guidance for applying a proportionate approach to the process of seeking consent (Health Research Authority, 2017) and researchers will be mindful and adapt their approach by slowing the process down to enable people with dementia seeking post-diagnostic support via a Recovery College dementia course to make an informed decision.

Once dates for the research course are identified and agreed, researchers will plan to attend and observe sessions. All in the room will be reassured that researchers are not looking at individuals’ ‘performance’, but rather looking at how the course is experienced by everyone in the room. In the interests of data transparency and inclusion, everyone involved will be given the opportunity to view researchers ethnographic record sheets if people so wish.

Following the course, researchers will invite co-producers and attendees to take part in individual one-to-one interviews and/or focus groups which will be arranged individually by telephone and/or email following the end of the course.

Supporting information for the initial process for obtaining valid informed consent We will provide each site with Participant Information Sheets before each stage of consent and data collection. Participant Information Sheets will make clear that participation is voluntary and that participants will have opportunities to discuss the study, ask questions and ask for further information before we ask individuals to give their informed consent. We will use a variety of methods for recording consent, according to the needs and wishes of each individual, either via post and telephone, or using Microsoft Teams/Zoom to record the completion of the consent form. Verbal consent will also be confirmed on each occasion we meet people.

Supporting people experiencing fluctuating mental capacity to participate if they wish Most people diagnosed in NHS memory services are likely to have mild to moderate dementia symptoms in the post-diagnostic phase. We are aware that for people living with a progressive cognitive impairment, fluctuating mental capacity can affect a person day-to-day, hour-to-hour and moment-to-moment, and that these experiences are different for each person. People with dementia may also experience difficulties communicating (both verbal and written) and responding to questions due to cognitive and/or sensory impairments. Participants who may lack capacity to consent will not be excluded, and consent will be treated as an ongoing process reviewed at each point of contact. Researchers will use a proforma for recording capacity assessment for people with dementia to give consent developed by the study Sponsor research department. In the event that a potential participant has fluctuating capacity, they will be asked what they would like to do if their capacity were to deteriorate, and if they have a trusted person we could speak to. Where people lack capacity, we will seek advice from a personal consultee (usually a person's family/friend supporter) on what the wishes and feelings of the person might be, and whether or not they should take part.

Process consent for ethnography in a group setting The case study site researchers responsible for obtaining valid informed consent, have extensive experience of delivering NIHR portfolio dementia studies using mixed methods in health and social care settings and will be observing the principles of process consent (Dewing, 2007) in dementia research and data collection at case study sites. They have extensive experience of using observational methods in research and clinical dementia care practice settings. Closely observing individuals at all stages of dementia for verbal and non-verbal signs of well and ill-being is central to interviewing, ethnographic and analysis activities, to address supporting participation when and where appropriate, and in a person-centred, inclusive and enabling way, to ensure all individuals viewpoints are heard. All stakeholders in the selected course will have been invited to participate and valid informed consent will have been established prior to the course start as detailed above. Adopting a process consent approach (Dewing, 2007) will guide practice during the observations by watching for any signs which may indicate a non-verbal withdrawal of consent by individuals. If this is the case, we won't stop the observations, but we will not record any further actions from the individual.

Ensuring information is accessible We will be flexible in the ways in which we communicate on the day/s of ethnography and individual interviews, for example providing information at different times, allowing extra time, introducing ourselves and drawing on our person-centred interpersonal skills. The PPI advisory group have shared with us some challenges of experiencing short-term memory difficulties in, for example, feeling less able to concentrate for usual-length meetings via Zoom. The group have helped improve accessibility of participant information sheets and recruitment materials. Where possible, we will also identify people who may have English language and/or reading and writing needs, and will engage

interpreters/translators where required, alongside the use of visual aids. For people who arrive on the day of the course session without prior informed consent, we will pragmatically ask family supporters if they are comfortable to act as a personal interpreter if needed. The research team are experienced in working with people with language needs, and neurological impairments, and have successfully used these methods in prior research studies to support inclusion and respect.

Process for recording consent the PPI advisory group suggest offering a range of choices to support this process after receiving and talking through the Participant Information sheet:

- On paper receiving a consent form by post and completing and returning via a pre-paid return envelope (using the Sponsor organisation FREEPOST service);
- On paper with a researcher face-to-face;
- Electronically via a secure Microsoft Forms link sent by email;
- Electronically by recording the consent part of the recruitment conversation online with a researcher via video platform.
- In person with local Clinical Research Network nurses

References

Health Research Authority. *Applying a proportionate approach to the process of seeking consent.* HRA Guidance. 2017. Available from: https://s3.eu-west-2.amazonaws.com/www.hra.nhs.uk/media/documents /applying-proportionate-approach-process-seeking-consent_R3gbJKn.pdf [Accessed 02nd August 2022].

Dewing J. Participatory research: a method for process consent with persons who have dementia. *Dementia*. 2007;6(1):11–25.

Flowchart illustrating points of consent activity:

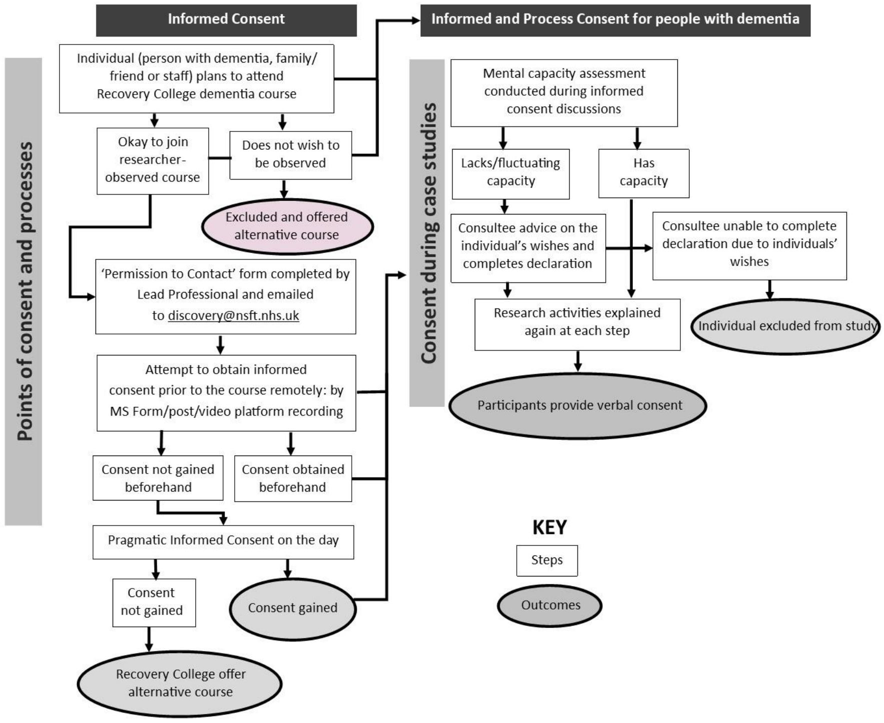

Supplement: Supplementary data [file bmjopen-2023-078248supp002.pdf]
