# Supplementary material for: Protocol for a realist evaluation of Recovery College dementia courses: understanding coproduction through ethnography
Source: BMJ Open. 2023 Dec 7;13(12):e078248. doi: 10.1136/bmjopen-2023-078248 (PMC10711820; doi:10.1136/bmjopen-2023-078248)
Supplement: Author's manuscript [file bmjopen-2023-078248.draft_revisions.pdf]

BMJ Open

BMJ Open is committed to open peer review. As part of this commitment we make the peer review history of every article we publish publicly available.

When an article is published we post the peer reviewers' comments and the authors' responses online. We also post the versions of the paper that were used during peer review. These are the versions that the peer review comments apply to.

The versions of the paper that follow are the versions that were submitted during the peer review process. They are not the versions of record or the final published versions. They should not be cited or distributed as the published version of this manuscript.

BMJ Open is an open access journal and the full, final, typeset and author-corrected version of record of the manuscript is available on our site with no access controls, subscription charges or pay-per-view fees (http://bmjopen.bmj.com).

If you have any questions on BMJ Open's open peer review process please email info.bmjopen@bmj.com

BMJ Open

Protocol for a realist evaluation of Recovery College dementia courses: Understanding co-production through ethnography

Journal:	*BMJ Open*
Manuscript ID	bmjopen-2023-078248
Article Type:	Protocol
Date Submitted by the Author:	27-Jul-2023
Complete List of Authors:	Birt, Linda; University of East Anglia, School of Health Sciences<br>West, Juniper; Norfolk and Suffolk NHS Foundation Trust, Research and Development<br>Poland, Fiona; University of East Anglia, School of Health Sciences<br>Wong, Geoff; Oxford University, Nuffield Department of Primary Care Health Sciences<br>Handley, Melanie; University of Hertfordshire, CRIPACC<br>Litherland, Rachael; Innovations in Dementia<br>Hackmann, Corinna; Norfolk and Suffolk NHS Foundation Trust, Research and Development<br>Moniz-Cook , Esme ; University of Hull, Faculty of Health Sciences,Department of Psychological Health and Well Being<br>Wolverson, Emma; University of Hull, Faculty of Health Sciences<br>Teague, Bonnie; Norfolk and Suffolk NHS Foundation Trust, Research<br>Mills, Ruth; Norfolk and Suffolk NHS Foundation Trust, Older People's Services<br>Sams, Kathryn; Norfolk and Suffolk NHS Foundation Trust, Older People's Services<br>Duddy, Claire; University of Oxford, Nuffield Department of Primary Care Health Sciences<br>Fox, Chris; Norwich Medical School, Department of Psychological Sciences; University of Exeter,
Keywords:	Dementia, Patient Participation, Old age psychiatry < PSYCHIATRY, QUALITATIVE RESEARCH

SCHOLARONE™
Manuscripts

BMJ Open Protocol Title Page v5 14.06.2023

Protocol for a realist evaluation of Recovery College dementia courses: Understanding co-production through ethnography

Authors

Linda Birt 1) School of Health Sciences, University of East Anglia https://orcid.org/0000-0002-4527-4414

Juniper West 2) Research and Development, Norfolk and Suffolk NHS Foundation Trust https://orcid.org/0000-0003-0802-7243

Fiona Poland 1) https://orcid.org/0000-0003-0003-6911

Geoffrey Wong 3) Nuffield Department of Primary Care Health Sciences, University of Oxford https://orcid.org/0000-0002-5384-4157

Melanie Handley 4) Centre for Research in Public Health & Community Care, University of Hertfordshire https://orcid.org/0000-0002-8037-5042

Rachael Litherland 5) Innovations in Dementia CIC, Exeter https://orcid.org/0009-0003-9747-6207

Corinna Hackmann 2) https://orcid.org/0000-0002-4940-6998

Esme Moniz-Cook 6) Faculty of Health Sciences, University of Hull https://orcid.org/0000-0002-7232-4632

Emma Wolverson 6) + 7) Research and Publications, Dementia UK, London, UK. https://orcid.org/0000-0002-5789-7862

Bonnie Teague 2) + 8) Department of Clinical Psychological and Psychological Sciences, University of East Anglia https://orcid.org/0000-0002-3248-2257

Ruth Mills 9) Older People's Services, Norfolk and Suffolk NHS Foundation Trust

Kathryn Sams 9)

Claire Duddy 10) Nuffield Department of Population Health, University of Oxford https://orcid.org/0000-0002-7083-6589

Christopher Fox 11) Medical School, University of Exeter + 9) https://orcid.org/0000-0001-9480-5704 on behalf of the DiSCOVERY study team.

Corresponding author Linda Birt linda.birt@uea.ac.uk

Acknowledgements *We would like to thank the DiSCOVERY Partners in Research (Patient and Public Involvement) advisory group who are actively contributing at all stages of this project, guiding the researchers to always consider actions from the perspectives of people living with dementia. Special thanks to Partner in Research Martyn Gardener who worked with the researchers to defend the application at the ethics committee meeting. Thanks to members of the memory services and Recovery College staff advisory group. Thanks also to the wider DiSCOVERY study team: Charlotte Wheeler, Leanne Hague, Thomas Rhodes, Maria*

For peer review only - http://bmjopen.bmj.com/site/about/guidelines.xhtml

Sanchez and Robert Kelly. Fiona Poland and Linda Birt's time has, in part, been supported by the NIHR East of England Applied Research Collaboration.

Author contributions

JW led on writing the protocol supported by all authors, and all authors contributed to protocol conception and design; LB is an expert on ethnographic methods; FP is an expert on social research methodology and community participative methods; GW is an experienced realist researcher; MH led the realist review which supported the development of this study; RL is the Patient and Public Involvement lead supporting people with dementia as stakeholders and overseeing co-production; BT contributed to protocol development and advised on issues relating to health inequalities; CF contributed to design and evaluation; LB and JW wrote the first draft of the article. All authors made substantial contributions to later versions.

Funding statement This study is funded by the National Institute for Health Research (NIHR) [NIHR Health and Social Care Delivery Research NIHR131676, 2022-2024]. The views expressed are those of the author(s) and not necessarily those of the NIHR or the Department of Health and Social Care.

This project is affiliated with the Inclusive Involvement in Research for Practice-led Health and Social Care within the NIHR East of England, which has supported Poland and Birt's time on this study. It develops work undertaken by West in her Collaboration for Leadership in Applied Health Research & Care (CLAHRC) East of England fellowship.

Conflicts of interests The authors declare no conflicting interests.

Keywords dementia, post-diagnostic support, recovery, co-production, peer support, realist evaluation, ethnicity, culture, intersectionality.

Word count 3044

For peer review only

Abstract

Introduction

Support following a dementia diagnosis in the UK is variable. Attending a Recovery College course with and for people with dementia, their supporters and healthcare professionals (staff), may enable people to explore and enact ways to live well with dementia. Recovery Colleges are established within mental health services worldwide, offering peer-supported short courses co-produced in partnership between staff and people with lived experience of mental illness. The concept of recovery is challenging in dementia narratives, with little evidence of how the Recovery College model could work as a method of post-diagnostic dementia support.

Methods and analysis

Using a realist evaluation approach, this research will examine and define what works, for whom, in what circumstances and why, in Recovery College dementia courses. The ethnographic study will recruit five case studies from NHS mental health Trusts across England. Sampling will seek diversity in new or long-standing courses, delivery methods and demographics of population served. Participant observations will examine course co-production. Interviews will be undertaken with people with dementia, family and friend supporters and staff involved in co-producing and commissioning the courses, as well as people attending. Documentary materials will be reviewed. Analysis will use a realist logic of analysis to develop a programme theory containing causal explanations for outcomes, in the form of context-mechanism-outcome-configurations, at play in each case.

Ethics and dissemination

The study received approval from Coventry & Warwickshire Research Ethics Committee (22/WM/0215). Ethical concerns include not privileging any voice, consent for embedded observational fieldwork with people who may experience fluctuating mental capacity, and balancing researcher 'embedded participant' roles in publicly-accessible learning events. Drawing on the realist programme theory, two stakeholder groups, one people living with dementia and one staff will work with researchers to co-produce resources to support co-producing Recovery College dementia courses aligned with post-diagnostic services.

Registration ISRCTN90339241

Strengths and Limitations

1. This study is the first to examine post-diagnosis Recovery College dementia courses run within NHS mental health Trusts.
2. The data collection methods have been discussed and agreed with a stakeholder group of people living with dementia.
3. Using a realist approach has the potential to provide understanding on contextual factors which affect outcomes.

For peer review only

4. To avoid privileging sub-groups the variety of people involved in co-producing and co-delivering Recovery College dementia courses such as staff, people with dementia and family supporters, will be analysed separately and then combined to provide a full picture.
5. Access to case sites may be limited due to post COVID-19 impacts on current provision of Recovery College dementia courses.

Introduction

Receiving and adjusting to a diagnosis of dementia is often life-changing for the person and their family, bringing many uncertainties, compounded by social stigma (1). The progressive nature of dementia in limiting communication and cognition brings anxieties for people trying to preserve identity and confidence in roles, relationships and interactions, and the person and their family may experience shame associated with self-stigma (2, 3). The post-diagnosis period is critical to adjustment (4) however post-diagnosis support globally varies enormously (5).

In the United Kingdom (UK) most people with dementia are assessed and diagnosed within specialist National Health Service (NHS) mental health provided memory services following a referral from their General Practitioner. Following a diagnosis of dementia, people should be offered support tailored to meet their individual needs (5, 6, 7). Some people do receive such support, however there is significant variability in the quality, duration and eligibility of post-diagnostic support across the UK, and what support there is, generally remains poorly defined or evidenced (7, 8).

One form of post-diagnostic support is through Recovery College courses. Recovery Colleges, delivered through NHS mental health Trusts and community organisations, support people holistically through individual mental health recovery by focusing on peer-led education and training. This is designed to complement existing clinical interventions and care (9). The ethos of the Recovery College model is to improve outcomes for people beyond a narrow focus on symptom reduction, to help people rebuild meaningful, satisfying lives, despite limitations caused by mental health difficulties (10, 11). Courses may be delivered either as a standalone session or across several sessions. All courses are co-designed and co-delivered - co-produced – by people with lived experience and healthcare professionals (staff). People are signposted to self-select and enrol, rather than being referred. In general, any service user, their supporters, or staff member can enrol on any course although this can differ between Recovery Colleges. All attendees are considered ‘students’, as Recovery Colleges aim to use language to empower their attendees and place all at an equal level.

Increasingly Recovery Colleges are co-producing courses specifically about living well with dementia. Recovery Colleges’ courses and content may vary, however they systematically reference and apply a robust conceptual framework which includes five domains of personal recovery - CHIME: Connectedness, Hope, Identity, Meaning and Empowerment (12). See supplementary file 1 for further details of the Recovery College model. Such courses align with the strategic health service aim to empower people living with dementia and their supporters to not only manage their symptoms but also to live positively or ‘well’ with dementia (13). Nonetheless, even when there is broad acceptance of a recovery ‘approach’ to dementia to support finding ways of living well alongside the diagnosis (14, 15), the use of the word ‘recovery’ used together with dementia does not appear to be acceptable to all staff working in memory services (15). The term requires further

exploration critically foregrounding the perspectives of people with lived experience among whom the mental health personal recovery approach originated (16).

The provision of courses for people living with dementia within the Recovery College model is variable across the UK (17). The impact of the COVID-19 pandemic on delivery of Recovery College dementia courses also appears significant with some courses ceasing and others moving to online delivery, although latterly there is a move back to face-to-face or hybrid delivery methods (15). Little is known about how the recovery model within the Recovery College works for people with dementia. Evaluation is needed to determine what works for who, how and when and therefore this ethnographic study is grounded within a wider project which draws on realist evaluation approaches.

Aims and objectives

This research project will use participant observation, interviews and review of documentary evidence across five case sites to examine what happens between people living with dementia, their family and friend supporters and staff. Interpreting data through the lens of a realist approach will enable us to address questions about why and how and in which way dementia courses are co-produced and the outcomes for peer tutors and those attending the courses.

Research Question

'What factors support the co-production and successful delivery of Recovery College dementia courses: what works, for whom, in what contexts and why?'

Methods and Analysis

Study design

We will conduct a realist evaluation (18) using a case study design to develop a realist programme theory, to understand how mental health service-delivered Recovery College courses currently lead to intended and unintended outcomes for people living with dementia, their family and friend supporters and staff. This work is the second step of a research project called DiSCOVERY (reference NIHR131676, 01 January 2022 to 31 December 2024) Learning from this research will support understandings on the implementation of a complex health service provision in different social and service contexts. It is likely to provide knowledge on how to optimise future course development and implementation on a national level.

The initial programme theory was developed from a realist review (manuscript under preparation). A case study approach is appropriate for understanding tightly bounded contexts, by place, time and actors, within naturally occurring events (19). Here a case is a Recovery College course offered to people following a diagnosis of dementia in English NHS mental health Trusts. The case is bounded by the time from beginning of co-production through delivery to evaluation of a single course. The place is either through online meeting or in person and the actors are any NHS staff involved in supporting Recovery Colleges; staff, people with dementia and family or friend supporters involved in the commissioning, co-production, delivery or evaluation of the course and any person with dementia, supporters or staff who attend the course. Data collection is ethnographic observation and questions, realist interviews and review of documents relating to the courses.

For peer review only

Patient and public involvement - co-produced research

In accordance with the ethos of co-production inherent in Recovery Colleges, in this study we are working throughout the whole programme of research with a diverse Patient and Public Involvement (PPI) - the Partners in Research - advisory group consisting of nine individuals; seven people living with dementia and two people who identify as family carers/supporters. The group's activity is supported by Innovations in Dementia (http://www.innovationsindementia.org.uk/) and the membership has the potential to be flexible as people's wish to be involved may change over the 36 months of the study. The advisory group have been instrumental from the very beginning in developing the research funding application through to refining the aim and considering the practical and ethical challenges of undertaking ethnography in this context. Through a regular series of online meetings, we are co-producing and refining our initial programme theory with our two key stakeholder advisory groups: PPI and staff from memory services and/or involved in Recovery College dementia courses. The PPI advisors in particular are involved in co-producing accessible materials for results dissemination throughout each study work package.

Recruitment

We plan to recruit up to five case sites across England sampling for the following case characteristics: are running a dementia course in person or online, have experience of delivering Recovery College courses in dementia or are delivering a first course. Case sites will additionally be approached to discuss aspects of cultural inclusion within their recovery course thinking, as reflected within the specific needs and diversity of their local populations. Heterogeneity in cases is important to understand a range of experiences to inform later work to co-produce relevant guidance documents. 'A case' needs to meet two requirements:

i. NHS mental health Trust provided Recovery College course on living well with dementia post-diagnosis in England;
ii. Availability of all stakeholder groups involved in the Recovery College dementia course, namely staff, people with dementia, family/friend supporters involved in **a.** co-production and/or delivery and **b.** as course attendees

Sample

A purposive sample will include characteristics highlighted through our realist review as most relevant for developing the programme theory. We aim to reflect the diversity of the populations Recovery Colleges serve and the types of courses they provide, to enable analyses to reflect and illuminate developments in different contexts. Based on our realist review these are:

Inclusion Criteria

- NHS Recovery Colleges which support people with dementia accessing memory services, including people from diverse ethnic and cultural local populations, socioeconomic and deprivation indices, urban/rural population mix;
- Recovery Colleges offering virtual or hybrid as well as traditional face-to-face courses;
- Courses offered by different staff groups i.e. psychologists, nurses, doctors, occupational therapists, support workers/assistant practitioners;
- Courses that involve both observable co-production and co-delivery of the course.

Exclusion criteria

- Courses provided for or predominantly attended by family carers or staff;
- Courses not co-produced with people with dementia.

A Recovery College dementia course co-production group typically ranges from 2 - 4 people; we plan to recruit and interview all who consent; across 5 case sites this will yield a sample of around n=15 individuals. These will include people living with dementia, family supporters and staff all involved in co-production and co-delivery of the course. Staff could be nurses, psychologists, support workers/assistant practitioners, doctors, occupational therapists, other allied healthcare professionals or service support staff.

The usual number of attendees at Recovery College dementia courses ranges from 8 - 20 people. Across 5 case sites this would yield a recruitment pool of between 40-100 people. We will purposively sample to recruit n=3-5 people per site ranging from people living with dementia, family/friend supporters and staff.

To examine service provision, we aim to recruit n=1-2 managers per site, who could be memory service managers/clinical team leaders or Recovery College leads/representative staff supporting Implementing Recovery for Organisational Change (ImROC) work in NHS mental health Trusts, giving a sample of 5-10 managers.

Consent

Once confirmation of capacity and capability has been received from each participating NHS mental health Trust and a Lead professional identified, those people co-producing the course will be consented into the study. Then as people enrol on the Recovery College dementia course, they will be advised of the planned research and invited to find out more about the study. PPI advisors have suggested the following words to use for the initial approach to each person booking a place on the course: *"Would you mind if two researchers join your course group?"*. At this point for people who do not wish to be involved in the research, the Lead Professional will coordinate their individual booking to alternative courses/dates. See supplementary file 2 for further details on valid informed consent processes.

Data collection

Within each case we will collect three types of data: **1** focused ethnography, **2** realist interviews and **3** documentary evidence. Data collection will take place over 18 months to provide time to capture data across one whole course.

1 Focused ethnography

Focused ethnography methods use short, intensive, linked field visits (20, 21). This method is pragmatically, ethically and methodologically appropriate here in not imposing an unduly heavy burden on people. Two case study site researchers will carry out ethnographic observations, potentially with a PPI advisory group member as a co-researcher. The co-researcher may make notes if they chose but they will have a reflective conversation with the researchers after each observation event. All will be situated as 'embedded participants' among course attendees. Each case study will entail a minimum of four hours observation but depending on length of preparation and course, this may be up to 14 hours. The findings derived from our realist review and subsequent PPI and staff advisory group discussions will further direct the focus for observations. A post-session debrief and reflection period held with the course co-producers/facilitators will explore whether having

researchers as embedded participants changed the dynamics or behaviours in the room. Researchers will transcribe their handwritten field notes up as soon as possible after the observation, within 72 hours. Materials relevant to the observation such as floor plans or course materials will be stored alongside observations.

2 Realist interviews (22) After the course is delivered, we will conduct individual semi-structured qualitative interviews with course co-producers and attendees as well as NHS managers in memory services or Recovery College staff. Interviews will be undertaken within two weeks of the course finishing and may be face-to-face or virtual depending on the participants preference. They will be recorded and professionally transcribed verbatim. Interview topic guides were developed based on the findings of the realist review and together with the PPI advisory group. Using focused ethnography initially will provide the researchers with detailed in-depth understanding of what happens in practice, and this will then inform interviewing to interrogate areas of tension, complexity and uncertainty. A second interview will be offered to course co-producers and attendees who wish to speak specifically about meeting the needs of ethnic and cultural minority groups in their courses. A brief summary of key points from the interview transcripts will be made available on request for participants to help people, particularly participants with dementia, to revisit their conversation, as was suggested by the PPI advisory group. This provides a chance for participants to change or add anything to the summary.

3 Documentary evidence We will review relevant documents such as course materials and evaluations to provide context and some triangulation with observations and interviews. For example, whether anonymised evaluations resonate with experiences discussed in interviews.

Data analysis

All relevant data will be uploaded into NVivo (23), a software package that supports qualitative data analysis. An iterative approach to analysis will enable emerging ideas to be refined and further explored in later case studies. We will analyse data collected using a realist logic (18). Data coding will be deductive (informed by our initial programme theory developed from the realist review), inductive (derived from the collected data) and retroductive (making inferences about mechanisms based on interpretations of our data to infer underlying causal processes). Questions on interpretations and judgement will guide the focus of analysis as outlined in **Table 1**.

Table 1. Questions to direct analysis of data from ethnography, interviews and documentary evidence

Relevance	Is source text section relevant to programme theory development?
Interpretation of meaning	If yes, does text content provide data that might be interpreted as functioning as **context, mechanism or outcome (C, M or O)**?
Interpretation and judgement about CMO configurations	For data that functions as C, M or O, which **CMO configuration (CMOC)** (partial or complete) does it belong to?<br>Can additional data informing this particular CMOC be found either in this source or other sources? If so which sources? How does this CMOC relate to other already-developed CMOCs?
Interpretations and judgements about programme	How does this particular (full or partial) CMOC relate to the programme theory?<br>What data from our sources inform how the CMOC relates to the programme theory? If yes, which sources provide which data?<br>Does the construction of this specific CMOC and any supporting data,

theory	require the programme theory to be changed?

Using a case study approach means that interpretations such as inferred mechanism of outcome can be examined and compared across cases to better explain why and how observed outcomes occur. We can compare 'successful' with 'less successful' cases of Recovery College courses, to examine how specific contexts or mechanisms have influenced outcomes observed. When using the questions in **Table 1**, we will also use the following forms of reasoning to make sense of the data:

- Juxtaposing: for example, where data about behaviour change in one source enables insights into data about outcomes in another source.
- Reconciling: where data differs between apparently similar circumstances (i.e. interviews and documentary review), it is appropriate to seek potential explanations for why these differences having occurred.
- Adjudicating: in terms of methodological strengths or weaknesses of the data sources.
- Consolidating data: where outcomes differ in particular contexts, constructing an explanation of how and why these outcomes occur differently.

In summary, the aim of this realist evaluation data collection and analysis is to specify theoretical claims that are grounded in data and that may be made transferable to real world contexts. Examining interview, observation and document data alongside each other will enable us to identify and apply differing perspectives and understandings, to refine the programme theory, in relation to experiences and discussions of dementia.

Data management

All confidential data such as consent forms and other study documentation will be archived securely for a period of 10 years in accordance with sponsor policy. Interview recordings will be transcribed and anonymised then destroyed. All data will have any identifying features removed. Data for analysis will be managed in NVivo (23). To support the development of the programme theory, all study data, such as interview transcripts, observations, notes from stakeholder and researcher analysis meetings are stored in a central file and ordered by date and type. Non-identifiable data sets will be made available to other researchers at reasonable request. Data management and storage will comply with UK General Data Protection Regulations (24).

review only

Ethics and Dissemination

An important ethical concern in this study is gaining and monitoring consent from people living with dementia who may have fluctuating capacity. We will use the principles of process consent (25) - see supplementary file 2. Ethical approval was given by Coventry & Warwickshire Research Ethics Committee (22/WM/0215). This is a committee flagged for expertise in studies in dementia. A PPI advisory group member living with dementia attended the review meeting with the research team. The person found this an interesting experience and were active in directly addressing questions from the ethical review committee.

Learning from this research will be used in the following study work package where we will co-produce guidance and resources for groups planning or currently delivering Recovery College courses in dementia. Additional findings and learning will be disseminated through peer-reviewed

publications, conferences, lay reports and via our website https://www.nsft.nhs.uk/discovery-study/.

Discussion

This ethnography draws on realist evaluation approaches to analyse, interpret and judge theoretical implications of data from naturally-occurring Recovery College courses in dementia. It is likely to be the first study to report rigorously analysed findings, using a realist approach, from what happens during the co-production and co-delivery of such courses to include the perspective and actions of people living with dementia, their family and friend supporters and healthcare staff, and how they affect each other in the context of each setting. This means identifying, recording and integrating outcomes and mechanisms for the course attendees and not only for the peer tutors and staff. This evaluation of Recovery College dementia courses will provide comprehensive evidence on their place within post-diagnostic support pathways in dementia, essential for implementing evidence-based practice in Recovery College dementia courses.

References

(1) Rewerska-Juśko M, Rejdak K. Social Stigma of People with Dementia. *J Alzheimers Dis*. 2020;78(4):1339–1343. doi: 10.3233/JAD-201004. PMID: 33185610.

(2) Herrmann LK, Welter E, Leverenz J et al. A Systematic Review of Dementia-related Stigma Research: Can We Move the Stigma Dial?. *Am. J. Geriatr. Psychiatry*. 2018;26(3):316–331.

(3) Aldridge H, Fisher P, Laidlaw K. Experiences of shame for people with dementia: An Interpretative Phenomenological Analysis. *Dementia*. 2019;18(5):1896–1911. doi: 10.1177/1471301217732430

(4) O'Shea E, Keogh F, Heneghan C. Post-Diagnostic Support for People with Dementia and their Carers (online). 2018. https://www.understandtogether.ie/news-and-events/news /Dementia-Post-diagnostic-Support-Literature-Review.pdf (Accessed 08 September 2020).

(5) Gauthier S, Webster C, Servaes S et al. 2022. World Alzheimer Report 2022: Life after diagnosis: Navigating treatment, care and support. London, England: Alzheimer's Disease International.

(6) Copland E, Hodge S, Clary L, Cartwright V. Memory Services National Accreditation Programme (MSNAP) Standards: Standards for Memory Services (online). 2018. https://www.rcpsych.ac.uk/docs /default-source/improving-care/ccqi/quality-networks/memory-clinics-msnap/msnap-standards-6th-edition-2018.pdf (Accessed 03 March 2021).

(7) Frost R, Walters K, Wilcock J et al. Mapping post-diagnostic dementia care in England: an e-survey. *J Integr Care*. 2020;29(1):22–36.

(8) Wheatley A, Bamford C, Brunskill G et al. Task-shifted approaches to post-diagnostic dementia support: a qualitative study exploring professional views and experiences. *BMJ Open*. 2020;10(9):1–8.

(9) Perkins R, Repper J, Rinaldi M, Brown H. Recovery Colleges. ImROC briefing 1 (online). 2012. https://imroc.org/wp-content/uploads/2022/04/1.Recovery-Colleges.pdf (Accessed 07 June 2023).

(10) Perkins R, Hill L, Daley S et al. 'Continuing to be me' – Recovering a life with a Diagnosis of Dementia. ImROC briefing 12 (online). 2013. https://imroc.org/resource/12-continuing-to-be-me-recovering-a-life-with-a-diagnosis-of-dementia/ (Accessed 07 June 2023).

For peer review only

(11) Perkins R, Meddings S, Williams S, Repper J. Recovery Colleges 10 Years On. ImROC briefing 15 (online). 2018. https://imroc.org/wp-content/uploads/2022/04/ImROC-Recovery-Colleges-10-Years-On.pdf (Accessed 07 June 2023).
(12) Leamy M, Bird V, Le Boutillier C et al. Conceptual framework for personal recovery in mental health: Systematic review and narrative synthesis. *Br J Psychiatry*. 2011;199(6):445–52.
(13) Department of Health. Living well with dementia: A National Dementia Strategy. 2009. Living Well With Dementia: a national dementia strategy - GOV.UK (www.gov.uk) (Accessed 01 March 2023).
(14) Hill L, Roberts G, Wildgoose J et al. Recovery and person-centred care in dementia: Common purpose, common practice? *Adv Psychiatr Treat*. 2010;16(4):288–98.
(15) Wolverson E, Hague L, West J, Teague B, Fox C, Birt L, Mills R, Rhodes T, Sams K, Moniz-Cook E. Building an understanding of UK Recovery College dementia courses: A national survey of Recovery College and memory services staff. *Work Older People*. 2023. In review.
(16) Perkins R, Repper J. Editorial Reclaiming Recovery. *Ment Heal Soc Incl*. 2021;25(1):1–6. doi: 10.1108/MHSI-02-2021-082
(17) Lowen C, Birt L, West J. Recovery colleges and dementia courses – a scoping survey. *Ment Heal Soc Incl.* 2019;23(4):166–72.
(18) Pawson R, Tilley N. Realistic Evaluation. London, SAGE Publications 1997.
(19) Yin RK. Case study research and applications: Design and methods. 6th ed. SAGE Publications 2017.
(20) Wall S. Focused Ethnography: A Methodological Adaptation for Social Research in Emerging Contexts. *Forum Qual Sozialforsch / Forum Qual Soc Res*. 2015;16(1):1–11.
(21) Knoblauch H. Focused Ethnography. *Forum Qual Sozialforsch / Forum Qual Soc Res.* 2005;6(3): 1-11.
(22) Manzano A. The craft of interviewing in realist evaluation. *Evaluation.* 2016;22(3):342–360. doi: 10.1177/1356389016638615
(23) QSR International Pty Ltd. (2020) NVivo (released in March 2020), https://www.qsrinternational.com/nvivo-qualitative-data-analysis-software/home
(24) Guide to the UK General Data Protection Regulation (UK GDPR) (online). 2018. https://ico.org.uk /for-organisations/guide-to-data-protection/guide-to-the-general-data-protection-regulation-gdpr/ (Accessed 08 June 2023).
(25) Dewing J. Participatory research: a method for process consent with persons who have dementia. *Dementia*. 2007;6(1):11–25.

- **Competing interests statement.**
- **Word Count:** 4,000 words. Should the word count exceed this number, please state this in the cover letter upon submission.

For peer review only

Supplementary file 1

Recovery Colleges

The first UK Recovery College was set up in 2009, growing to 85 by 2017 (Anfossi, 2017). The model has been replicated internationally (Perkins et al., 2018) with Recovery Colleges now in existence or developing in 22 countries (King & Meddings, 2019). Recovery College courses offer access to distinctive peer support both from co-producing courses and/or attending them (Sommer, Gill & Stein-Parbury, 2018). A national strategy to promote dementia peer support through developing learning networks also aims to empower people living with dementia to make choices in planning for the future (Department of Health, 2009). A 'recovery-focused', peer-support, adult learning approach is adopted and embedded within a strategic care delivery approach in mental health Trusts, to improve care outcomes beyond a narrow focus on symptom reduction, to help people rebuild meaningful, satisfying lives, despite limitations caused by mental health difficulties (Perkins et al., 2018). Recovery Colleges create adult learning environments which moderate power dynamics between service users and staff, to reduce stigma and increase attendees' sense of hope and empowerment (Meddings et al., 2014; Sommer, Gill & Stein-Parbury, 2018; Zabel et al., 2016). Attendees report developing novel coping strategies, improving self-worth, wellbeing and quality of life (Meddings et al., 2014; Rinaldi, Marland & Wybourn, 2011; Secker & Wilson, 2014; Zucchelli & Skinner, 2013).

A typical adult mental health Recovery College offers courses on mental health and recovery, designed to increase attendees' knowledge, skills and confidence in self-management of their own mental health and wellbeing. Of note, what 'wellbeing' means for people with dementia may be very different, but has been defined as emotional (positive states), social (connections and belonging) and psychological (positive sense of self, going beyond and agency and purpose) with an overarching theme of 'valuing life', which differs to studies evaluating life satisfaction (Clarke et al., 2020). Courses range from one-off sessions to several sessions spread over a set number of weeks. All courses are co-produced and co-delivered (i.e. co-taught) by peer tutors - that is, people with lived/expert experience - and mental health staff, who also enrol on courses as attendees. Peer tutors prepare for their role through having training to teach and support, and receive supervision to ensure any sensitive issues can be supported effectively.
A theory of change model for Recovery Colleges has been co-developed within adult mental health contexts that identifies four mechanisms of change (Toney et al., 2018) empowering environment - opportunities for choices; shifting balance of power; enabling different relationships and connecting with peers; and facilitating personal growth through shared learning and strength-building. Outcomes were changes in the attendee including improved wellbeing, reinforced by life changes they could observe. This model is highly applicable to enabling desired outcomes for post-diagnostic support in dementia.

A 'recovery-focused', peer-support, adult learning approach is adopted and embedded within a strategic care delivery approach in mental health Trusts, to improve care outcomes beyond a narrow focus on symptom reduction, to help people rebuild meaningful, satisfying lives, despite limitations caused by mental health difficulties (Perkins et al., 2018). This approach is already flourishing in adult mental health services, encouraged since 2011 by the Department of Health commissioned 'Implementing Recovery through Organisational Change' (ImROC) collaborative https://imroc.org/. Working with mental health Trusts, ImROC founded Recovery Colleges which have rapidly become a core part of recovery-focused mental health services. As of 2017, there were 85 Recovery Colleges in the UK (77 in England, 2 in Scotland, 5 in Northern Ireland and 1 in Wales; Anfossi, 2017).

Recovery Colleges further have five key linked conceptual processes - the **CHIME** recovery framework – robustly developed to underpin the term 'recovery' in this context: **connecting** with others, inspiring **hope**, maintaining a positive **identity**, finding **meaning** in life outside of symptoms and **empowering** control over life and a focus on strengths (Leamy et al., 2011).

The CHIME Recovery framework, as operationalised through Recovery Colleges, has clearly relevant links with the NICE-recommended person-centred care framework for dementia (Leamy et al., 2011; Brooker, 2007), the Royal College of Psychiatrists Memory Services National Accreditation Programme (MSNAP; Copland et al., 2018) and the National Dementia Strategy objective to develop peer support and learning networks (Department of Health, 2009). Key domains for person-centred care are **valuing** people living with dementia and those (both informal family and friends and health and social care staff) who care for them; providing care that is **individualised**; understanding and acting from the **perspectives** of people living with dementia (which can reinforce connections, meanings and identities); and creating positive **social-psychological** environments (which can build hope and empowerment; Brooker, 2007). For dementia care to be person-centred, all these elements are needed and need to work together. Both frameworks require mental health services to continuously improve, and to evidence progress towards more meaningful care within each of these domains (Brooker, 2007; Leamy ey al., 2011,) yet person-centred principles are still poorly and inconsistently translated into practice. Applying these frameworks in memory post-diagnostic support services is important for keeping a whole person focus so as to value the identity, perspectives, strengths and needs of people living with dementia, and so to enable staff to share with patients more effectively care planning alongside understandings of personal recovery in adjusting to dementia.

Five recovery processes giving the acronym CHIME

Connectedness with others;
inspiring **Hope** and optimism about the future;
maintaining a positive **Identity**;
finding **Meaning** in life outside of symptoms;
and **Empowerment** with control over life and a focus on strengths. (Leamy et al., 2011)

References

Anfossi A. The current state of Recovery Colleges in the UK : final report. *ImROC.* 2017.
Brooker D. *Person-centred Dementia Care: Making services better.* London: Jessica Kingsley; 2007.
Clarke C, Woods B, Moniz-Cook E, Mountain G, Øksnebjerg L, Chattat R, Diaz A, Gove D, Vernooij-Dassen M, Wolverson E. Measuring the well-being of people with dementia: a conceptual scoping review. *Health and quality of life outcomes.* 2020;18(1), 1–14
Copland E, Hodge S, Clary L, Cartwright V. *Memory Services National Accreditation Programme (MSNAP) Standards: Standards for Memory Services*. London; 2018. Available from: https://www.rcpsych.ac.uk/docs /default-source/improving-care/ccqi/quality-networks/memory-clinics-msnap/msnap-standards-6th-edition-2018.pdf [Accessed 3rd March 2021].
Department of Health. *Living well with dementia: A National Dementia Strategy.* 2009. Available from: https://assets.publishing.service.gov.uk/government/uploads/system/uploads/attachment_data/file /168220/dh_094051.pdf [Accessed 10th December 2019].
King T, Meddings S. Survey identifying commonality across international Recovery Colleges. *Ment Heal Soc Incl.* 2019;23(3):121–8.

For peer review only

Leamy M, Bird V, Le Boutillier C, Williams J, Slade M. Conceptual framework for personal recovery in mental health: Systematic review and narrative synthesis. *Br J Psychiatry*. 2011;199(6):445–52.
Meddings S, Byrne D, Barnicoat S, Campbell E, Locks L. Co-Delivered and Co-Produced: Creating a Recovery College in Partnership. Jce . Vol 9 (1); pp16-25. *J Ment Heal Training, Educ Pract.* 2014;9(1):16–25.
Meddings S, McGregor J, Roeg W, Shepherd G. Recovery Colleges: Quality and outcomes. *Ment Heal Soc Incl*. 2015;19(4):212–21.
Perkins R, Meddings S, Williams S, Repper J. Recovery Colleges 10 Years On. *ImROC.* 2018
Rinaldi M, Marland M, Wybourn S. *Annual Report 2011 – 2012 South West London Recovery College*. 2012. Available from: http://rfact.org.au/wp-content/uploads/2015/05/SW-London-Recovery-College-evaluation-2011_12-v1-0.pdf [Accessed 12th February 2020].
Secker J, Wilson C. *Evaluation of the mid Essex Recovery College, October – December 2013. 2014.* Anglia Ruskin University, 2014.
Sommer J, Gill K, Stein-Parbury J. Walking side-by-side: Recovery Colleges revolutionising mental health care. *Ment Heal Soc Incl*. 2018;22(1):18–26.
Toney R, Elton D, Munday E, Hamill K, Crowther A, Meddings S, et al. Mechanisms of action and outcomes for students in recovery colleges. *Psychiatr Serv.* 2018;69(12):1222–9.
Zabel E, Donegan G, Lawrence K, French P. Exploring the impact of the recovery academy: a qualitative study of Recovery College experiences. *J Ment Heal Training, Educ Pract*. 2016;11(3):162–71.
Zucchelli FA, Skinner S. Central and North West London NHS Foundation Trust's (CNWL) Recovery College: the story so far. *Ment Heal Soc Incl*. 2013;25

For peer review only

Supplementary file 3 Valid Informed Consent Processes

Process of obtaining consent from each co-production group The co-production group at each site will be contacted first and we aim to be as flexible and pragmatic as possible in recruiting and seeking informed consent from individuals in the group so we can begin observing co-production activities and documents as soon as possible prior to the researcher-observed course starting.

- Two case study researchers arrange an informal pre-course meeting with the co-production group to seek permission to contact for individual consent meeting/s
- Facilitate introductions, give information about the study, answer any questions, discuss and plan to join further meetings as ethnographic observers of the co-production process
- Gain understanding of practicalities around course publicity, set up and co-facilitation
- Discuss what types of documents and materials might be available for researchers to review

Process of obtaining consent from attendees Similarly we aim to be as flexible and pragmatic as possible in recruiting and seeking informed consent from all dementia course attendees. When people enrol on the Recovery College dementia course, they will be advised of the planned research and invited to find out more about the study. If at this point people do not wish to be involved in the research, the Lead Professional will coordinate their individual booking to alternative courses/dates.

If a person agrees in principle, the first line of obtaining consent will be for attendees who inform the case site Lead Professional they are planning to attend the course. The Lead Professional/site staff will provide the attendee with a participant information sheet. If agreed with the potential attendee, a 'permission to contact' form will be completed by the Lead Professional/site staff and sent securely via email to the study team via the DiSCOVERY study email address. The study team will attempt to obtain informed consent from all attendees, including healthcare professionals, people with dementia and their family supporters via remote methods prior to the day of data collection. If remote methods of informed consent are not possible, attendees will be offered the opportunity to speak to the study team in person prior to the course and provide informed consent on the day. However it is possible that people will attend the course without providing prior consent for taking part in the study. In those cases, attendees will be provided study information and offered the opportunity to take part in the research study immediately prior to the data collection commencing in accordance with HRA guidance for applying a proportionate approach to the process of seeking consent (Health Research Authority, 2017) and researchers will be mindful and adapt their approach by slowing the process down to enable people with dementia seeking post-diagnostic support via a Recovery College dementia course to make an informed decision.

Once dates for the research course are identified and agreed, researchers will plan to attend and observe sessions. All in the room will be reassured that researchers are not looking at individuals' 'performance', but rather looking at how the course is experienced by everyone in the room. In the interests of data transparency and inclusion, everyone involved will be given the opportunity to view researchers ethnographic record sheets if people so wish.

Following the course, researchers will invite co-producers and attendees to take part in individual one-to-one interviews and/or focus groups which will be arranged individually by telephone and/or email following the end of the course.

Supporting information for the initial process for obtaining valid informed consent We will provide each site with Participant Information Sheets before each stage of consent and data collection. Participant Information Sheets will make clear that participation is voluntary and that participants will have opportunities to discuss the study, ask questions and ask for further information before we ask individuals to give their informed consent. We will use a variety of methods for recording consent, according to the needs and wishes of each individual, either via post and telephone, or using Microsoft Teams/Zoom (REFREF) to record the completion of the consent form. Verbal consent will also be confirmed on each occasion we meet people.

Supporting people experiencing fluctuating mental capacity to participate if they wish Most people diagnosed in NHS memory services are likely to have mild to moderate dementia symptoms in the post-diagnostic phase. We are aware that for people living with a progressive cognitive impairment, fluctuating mental capacity can affect a person day-to-day, hour-to-hour and moment-to-moment, and that these experiences are different for each person. People with dementia may also experience difficulties communicating (both verbal and written) and responding to questions due to cognitive and/or sensory impairments. Participants who may lack capacity to consent will not be excluded, and consent will be treated as an ongoing process reviewed at each point of contact. Researchers will use a proforma for recording capacity assessment for people with dementia to give consent developed by the study Sponsor research department. In the event that a potential participant has fluctuating capacity, they will be asked what they would like to do if their capacity were to deteriorate, and if they have a trusted person we could speak to. Where people lack capacity, we will seek advice from a personal consultee (who may or may not be the person's family/friend supporter) on what the wishes and feelings of the person might be, and whether or not they should take part.

Process consent for ethnography in a group setting The case study site researchers responsible for obtaining valid informed consent, have extensive experience of delivering NIHR portfolio dementia studies using mixed methods in health and social care settings and will be observing the principles of process consent (Dewing, 2007) in dementia research and data collection at case study sites. They have extensive experience of using observational methods in research and clinical dementia care practice settings. Closely observing individuals at all stages of dementia for verbal and non-verbal signs of well and ill-being is central to interviewing, ethnographic and analysis activities, to address supporting participation when and where appropriate, and in a person-centred, inclusive and enabling way, to ensure all individuals viewpoints are heard. All stakeholders in the selected course will have been invited to participate and valid informed consent will have been established prior to the course start as detailed above. Adopting a process consent approach (Dewing, 2007) will guide practice during the observations by watching for any signs which may indicate a non-verbal withdrawal of consent by individuals. If this is the case, we won't stop the observations, but we will not record any further actions from the individual.

Ensuring information is accessible We will be flexible in the ways in which we communicate on the day/s of ethnography and individual interviews, for example providing information at different times, allowing extra time, introducing ourselves and drawing on our person-centred interpersonal skills. The PPI advisory group have shared with us some challenges of experiencing short-term memory difficulties in, for example, feeling less able to concentrate for usual-length meetings via Zoom (REF). The group have helped improve accessibility of participant information sheets and recruitment materials. Where possible, we will also identify people who may have English language and/or reading and writing needs, and will engage

interpreters/translators where required, alongside the use of visual aids. For people who arrive on the day of the course session without prior informed consent, we will pragmatically ask family supporters if they are comfortable to act as a personal interpreter if needed. The research team are experienced in working with people with language needs, and neurological impairments, and have successfully used these methods in prior research studies to support inclusion and respect.

Process for recording consent the PPI advisory group suggest offering a range of choices to support this process after receiving and talking through the Participant Information sheet:

- On paper receiving a consent form by post and completing and returning via a pre-paid return envelope (using the Sponsor organisation FREEPOST service);
- On paper with a researcher face-to-face;
- Electronically via a secure Microsoft Forms link sent by email;
- Electronically by recording the consent part of the recruitment conversation online with a researcher via video platform.
- In person with local Clinical Research Network nurses

References

Health Research Authority. *Applying a proportionate approach to the process of seeking consent.* HRA Guidance. 2017. Available from: https://s3.eu-west-2.amazonaws.com/www.hra.nhs.uk/media/documents /applying-proportionate-approach-process-seeking-consent_R3gbJKn.pdf [Accessed 02nd August 2022].

Dewing J. Participatory research: a method for process consent with persons who have dementia. *Dementia*. 2007;6(1):11–25.

For peer review only

Flowchart illustrating points of consent activity:

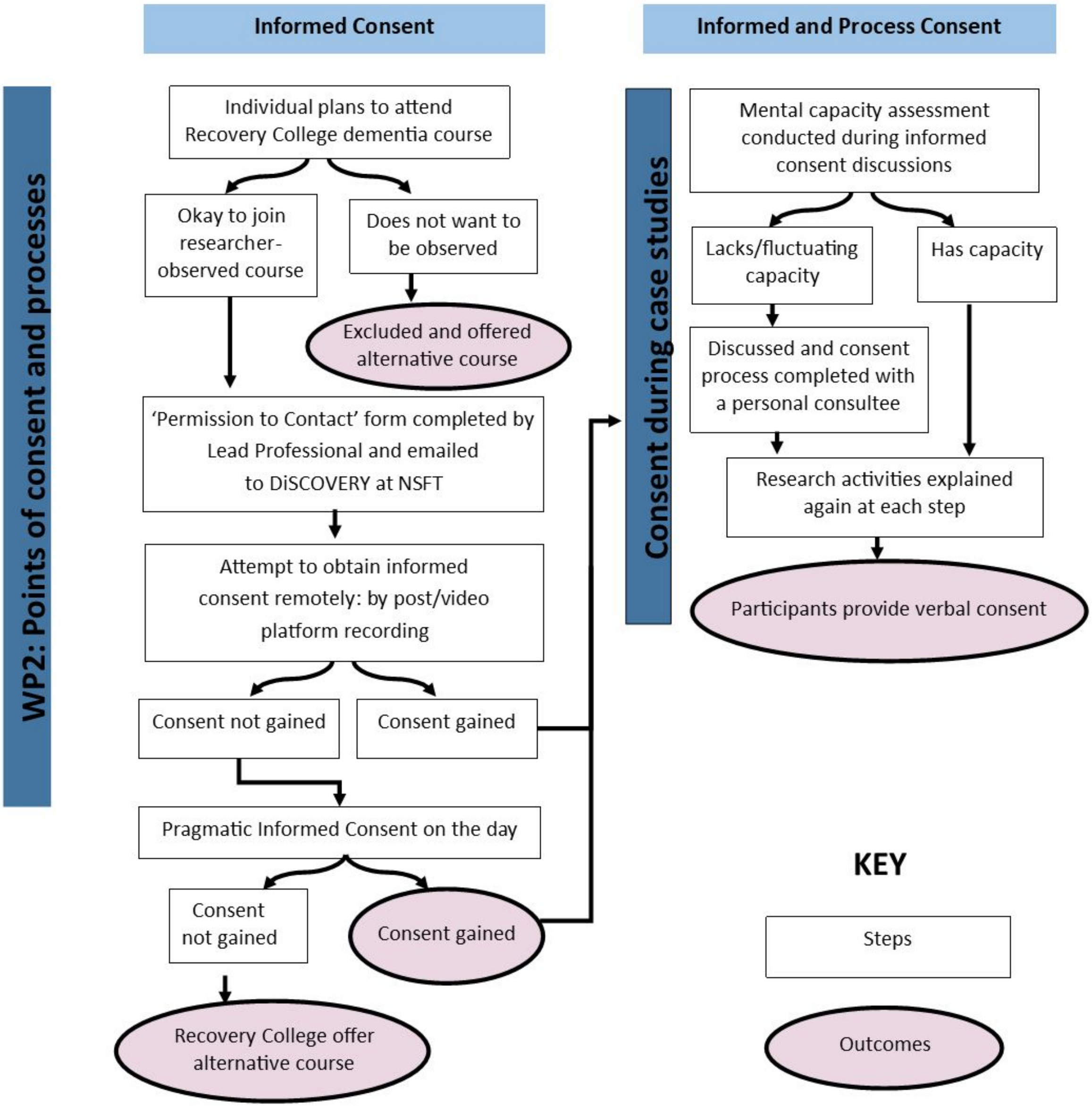

BMJ Open

Protocol for a realist evaluation of Recovery College dementia courses: Understanding co-production through ethnography

Journal:	*BMJ Open*
Manuscript ID	bmjopen-2023-078248.R1
Article Type:	Protocol
Date Submitted by the Author:	17-Oct-2023
Complete List of Authors:	Birt, Linda; University of East Anglia, School of Health Sciences<br>West, Juniper; Norfolk and Suffolk NHS Foundation Trust, Research and Development<br>Poland, Fiona; University of East Anglia, School of Health Sciences<br>Wong, Geoff; Oxford University, Nuffield Department of Primary Care Health Sciences<br>Handley, Melanie; University of Hertfordshire, CRIPACC<br>Litherland, Rachael; Innovations in Dementia<br>Hackmann, Corinna; Norfolk and Suffolk NHS Foundation Trust, Research and Development<br>Moniz-Cook , Esme ; University of Hull, Faculty of Health Sciences,Department of Psychological Health and Well Being<br>Wolverson, Emma; University of Hull, Faculty of Health Sciences<br>Teague, Bonnie; Norfolk and Suffolk NHS Foundation Trust, Research<br>Mills, Ruth; Norfolk and Suffolk NHS Foundation Trust, Older People's Services<br>Sams, Kathryn; Norfolk and Suffolk NHS Foundation Trust, Older People's Services<br>Duddy, Claire; University of Oxford, Nuffield Department of Primary Care Health Sciences<br>Fox, Chris; Norwich Medical School, Department of Psychological Sciences; University of Exeter,
**Primary Subject Heading**:	Health services research
Secondary Subject Heading:	Geriatric medicine, Mental health, Qualitative research
Keywords:	Dementia, Patient Participation, Old age psychiatry < PSYCHIATRY, QUALITATIVE RESEARCH

SCHOLARONE™
Manuscripts

BMJ Open Protocol Title Page

Protocol for a realist evaluation of Recovery College dementia courses: Understanding co-production through ethnography

Authors

Linda Birt 1) School of Health Sciences, University of East Anglia https://orcid.org/0000-0002-4527-4414

Juniper West 2) Research and Development, Norfolk and Suffolk NHS Foundation Trust https://orcid.org/0000-0003-0802-7243

Fiona Poland 1) https://orcid.org/0000-0003-0003-6911

Geoffrey Wong 3) Nuffield Department of Primary Care Health Sciences, University of Oxford https://orcid.org/0000-0002-5384-4157

Melanie Handley 4) Centre for Research in Public Health & Community Care, University of Hertfordshire https://orcid.org/0000-0002-8037-5042

Rachael Litherland 5) Innovations in Dementia CIC, Exeter https://orcid.org/0009-0003-9747-6207

Corinna Hackmann 2) https://orcid.org/0000-0002-4940-6998

Esme Moniz-Cook 6) Faculty of Health Sciences, University of Hull https://orcid.org/0000-0002-7232-4632

Emma Wolverson 6) + 7) Research and Publications, Dementia UK, London, UK. https://orcid.org/0000-0002-5789-7862

Bonnie Teague 2) + 8) Department of Clinical Psychological and Psychological Sciences, University of East Anglia https://orcid.org/0000-0002-3248-2257

Ruth Mills 9) Older People's Services, Norfolk and Suffolk NHS Foundation Trust

Kathryn Sams 9)

Claire Duddy 3) Nuffield Department of Population Health, University of Oxford https://orcid.org/0000-0002-7083-6589

Christopher Fox 11) Medical School, University of Exeter + 9) https://orcid.org/0000-0001-9480-5704 on behalf of the DiSCOVERY study team.

Corresponding author Linda Birt linda.birt@uea.ac.uk

Acknowledgements *We would like to thank the DiSCOVERY Partners in Research (Patient and Public Involvement) advisory group who are actively contributing at all stages of this project, guiding the researchers to always consider actions from the perspectives of people living with dementia. Special thanks to Partner in Research Martyn Gardener who worked with the researchers to defend the application at the ethics committee meeting. Thanks to members of the memory services and Recovery College staff advisory group. Thanks also to the wider DiSCOVERY study team: Charlotte Wheeler, Leanne Hague, Thomas Rhodes, Maria*

For peer review only

Sanchez and Robert Kelly. Fiona Poland and Linda Birt's time has, in part, been supported by the NIHR East of England Applied Research Collaboration.

Author contributions

JW led on writing the protocol supported by LB, FP, GW, MH, RL, CH, EMC, EW, BT, RM, KS, CD and CF, and all contributed to protocol conception and design; LB is an expert on ethnographic methods; FP is an expert on social research methodology and community participative methods; GW is an experienced realist researcher; MH led the realist review which supported the development of this study; RL is the Patient and Public Involvement lead supporting people with dementia as stakeholders and overseeing co-production; BT contributed to protocol development and advised on issues relating to health inequalities; CF contributed to design and evaluation; LB and JW wrote the first draft of the article. FP, GW, MH, RL, CH, EMC, EW, BT, CD and CF made substantial contributions to later versions.

Funding statement This study is funded by the National Institute for Health Research (NIHR) [NIHR Health and Social Care Delivery Research NIHR131676, 2022-2024]. The views expressed are those of the author(s) and not necessarily those of the NIHR or the Department of Health and Social Care.

This project is affiliated with the Inclusive Involvement in Research for Practice-led Health and Social Care within the NIHR East of England, which has supported Poland and Birt's time on this study. It develops work undertaken by West in her Collaboration for Leadership in Applied Health Research & Care (CLAHRC) East of England fellowship.

Conflicts of interests The authors declare no conflicting interests.

Keywords dementia, post-diagnostic support, recovery, co-production, peer support, realist evaluation, ethnicity, culture, intersectionality.

Word count 3812

For peer review only

Abstract

Introduction

Support following a dementia diagnosis in the UK is variable. Attending a Recovery College course with and for people with dementia, their supporters and healthcare professionals (staff), may enable people to explore and enact ways to live well with dementia. Recovery Colleges are established within mental health services worldwide, offering peer-supported short courses co-produced in partnership between staff and people with lived experience of mental illness. The concept of recovery is challenging in dementia narratives, with little evidence of how the Recovery College model could work as a method of post-diagnostic dementia support.

Methods and analysis

Using a realist evaluation approach, this research will examine and define what works, for whom, in what circumstances and why, in Recovery College dementia courses. The ethnographic study will recruit five case studies from NHS mental health Trusts across England. Sampling will seek diversity in new or long-standing courses, delivery methods and demographics of population served. Participant observations will examine course co-production. Interviews will be undertaken with people with dementia, family and friend supporters and staff involved in co-producing and commissioning the courses, as well as people attending. Documentary materials will be reviewed. Analysis will use a realist logic of analysis to develop a programme theory containing causal explanations for outcomes, in the form of context-mechanism-outcome-configurations, at play in each case.

Ethics and dissemination

The study received approval from Coventry & Warwickshire Research Ethics Committee (22/WM/0215). Ethical concerns include not privileging any voice, consent for embedded observational fieldwork with people who may experience fluctuating mental capacity, and balancing researcher ‘embedded participant’ roles in publicly-accessible learning events. Drawing on the realist programme theory, two stakeholder groups, one people living with dementia and one staff will work with researchers to co-produce resources to support co-producing Recovery College dementia courses aligned with post-diagnostic services.

Registration ISRCTN90339241

Strengths and Limitations

1. This study is the first to examine post-diagnosis Recovery College dementia courses run within NHS mental health Trusts.
2. The data collection methods have been discussed and agreed with a stakeholder group of people living with dementia.
3. Using a realist approach has the potential to provide understanding on contextual factors which affect outcomes.

For peer review only

4. To avoid privileging sub-groups the variety of people involved in co-producing and co-delivering Recovery College dementia courses such as staff, people with dementia and family supporters, will be analysed separately and then combined to provide a full picture.
5. Access to case sites may be limited due to post COVID-19 impacts on current provision of Recovery College dementia courses.

Introduction

Receiving and adjusting to a diagnosis of dementia is often life-changing for the person and their family, bringing many uncertainties, compounded by social stigma (1). The progressive nature of dementia in limiting communication and cognition brings anxieties for people trying to preserve identity and confidence in roles, relationships and interactions, and the person and their family may experience shame associated with self-stigma (2, 3). The post-diagnosis period is critical to adjustment (4) however post-diagnosis support globally varies enormously (5).

In the United Kingdom (UK) most people with dementia are assessed and diagnosed within specialist National Health Service (NHS) mental health provided memory services following a referral from their General Practitioner. Following a diagnosis of dementia, people should be offered support tailored to meet their individual needs (5, 6, 7). Some people do receive such support, however there is significant variability in the quality, duration and eligibility of post-diagnostic support across the UK, and what support there is, generally remains poorly defined or evidenced (7, 8).

One form of post-diagnostic support is through Recovery College courses. Recovery Colleges, delivered through NHS mental health Trusts and community organisations, support people holistically through individual mental health recovery by focusing on peer-led education and training. This is designed to complement existing clinical interventions and care (9). The ethos of the Recovery College model is not to frame the word 'recovery' as a clinical 'cure' for mental illness (10) but rather to improve outcomes for people beyond a purely medical model focus on symptom reduction, to help people rebuild meaningful, satisfying lives, despite limitations caused by mental health difficulties (10, 11). Courses may be delivered either as a standalone session or across several sessions. All courses are co-designed and co-delivered - co-produced – by people with lived experience and healthcare professionals (staff). People are signposted to self-select and enrol, rather than being referred. In general, any service user, their supporters, or staff member can enrol on any course although this can differ between Recovery Colleges. All attendees are considered 'students', as Recovery Colleges aim to use language to empower their attendees and place all at an equal level.

Increasingly Recovery Colleges are co-producing courses specifically about living well with dementia. Recovery Colleges' courses and content may vary, however they systematically reference and apply a robust conceptual framework which includes five domains of personal recovery - CHIME: Connectedness, Hope, Identity, Meaning and Empowerment (12). See supplementary file 1 for further details of the Recovery College model. Such courses align with the ~~strategic health service~~ national dementia strategy aim to empower people living with dementia and their supporters to not only manage their symptoms but also to live positively or 'well' with dementia (13). Nonetheless, even when there is broad acceptance of a recovery 'approach' to dementia to support finding ways of living well alongside the diagnosis (14, 15), the use of the word 'recovery' used together with dementia does not appear to be acceptable to all staff working in memory services (15). We

acknowledge concerns about the term ‘recovery’ in the context of dementia, and anticipate this study will contributing evidence towards the continuing wider debate (16,17) through exploring the perceptions of people with dementia and family supporters. The term requires further exploration critically foregrounding the perspectives of people with lived experience among whom the mental health personal recovery approach originated (18).

The provision of courses for people living with dementia within the Recovery College model is variable across the UK (19). The impact of the COVID-19 pandemic on delivery of Recovery College dementia courses also appears significant with some courses ceasing and others moving to online delivery, although latterly there is a move back to face-to-face or hybrid delivery methods (15). Little is known about how the recovery model within the Recovery College works for people with dementia. Evaluation is needed to determine what works for who, how and when and therefore this ethnographic study is grounded within a wider project which draws on realist evaluation approaches.

Aims and objectives

This research project will use participant observation, interviews and review of documentary evidence across five case sites to examine what happens between people living with dementia, their family and friend supporters and staff. Interpreting data through the lens of a realist approach will enable us to address questions about why and how and in which way dementia courses are co-produced and the outcomes for peer tutors and those attending the courses.

Research Question

‘What factors support the co-production and successful delivery of Recovery College dementia courses: what works, for whom, in what contexts and why?’

Objectives:

1. To examine if the characteristics of co producers leads to difference in outcomes for attendees
2. To examine if the content and delivery of course material leads to differences in outcomes for attendees
3. To examine if the recovery college has different outcomes for different types of attendees.

Methods and Analysis

Study design

We will conduct a realist evaluation (20) using a case study design to develop a realist programme theory, to understand how mental health service-delivered Recovery College courses currently lead to intended and unintended outcomes for people living with dementia, their family and friend supporters and staff. This work is the second step of a research project called DiSCOVERY (reference NIHR131676) with planned start and ends dates 01 January 2022 to 31 December 2024. Learning from this research will support understandings on the implementation of a complex health service provision in different social and service contexts. It is likely to provide knowledge on how to optimise future course development and implementation on a national level.

The initial programme theory was developed from a realist review (manuscript under preparation). A case study approach is appropriate for understanding tightly bounded contexts, by place, time and actors, within naturally occurring events (21). Here a case is a Recovery College course offered to

For peer review only

people following a diagnosis of dementia in English NHS mental health Trusts. The case is bounded by the time from beginning of co-production through delivery to evaluation of a single course. The place is either through online meeting or in person and the actors are any NHS staff involved in supporting Recovery Colleges; staff, people with dementia and family or friend supporters involved in the commissioning, co-production, delivery or evaluation of the course and any person with dementia, supporters or staff who attend the course. Data collection is ethnographic observation and questions, realist interviews and review of documents relating to the courses.

Patient and public involvement - co-produced research

In accordance with the ethos of co-production inherent in Recovery Colleges, in this study we are working throughout the whole programme of research with a diverse Patient and Public Involvement (PPI) - the Partners in Research - advisory group consisting of nine individuals; seven people living with dementia and two people who identify as family carers/supporters. Within the group two people have been involved in co-producing and delivering a recovery college dementia course. The group's activity is supported by Innovations in Dementia (http://www.innovationsindementia.org.uk/) and the membership has the potential to be flexible as people's wish to be involved may change over the 36 months of the study. The advisory group have been instrumental from the very beginning in developing the research funding application through to refining the aim and considering the practical and ethical challenges of undertaking ethnography in this context. Through a regular series of online meetings, we are co-producing and refining our initial programme theory with our two key stakeholder advisory groups: PPI and staff from memory services and/or involved in Recovery College dementia courses. The PPI advisors in particular are involved in co-producing accessible materials for results dissemination throughout each study work package.

Recruitment

We plan to recruit up to five case sites across England sampling for the following case characteristics: are running a dementia course in person or online, have experience of delivering Recovery College courses in dementia or are delivering a first course. Case sites will additionally be approached to discuss aspects of cultural inclusion within their recovery course thinking, as reflected within the specific needs and diversity of their local populations. Heterogeneity in cases is important to understand a range of experiences to inform later work to co-produce relevant guidance documents. 'A case' needs to meet two requirements:

i. NHS mental health Trust provided Recovery College course on living well with dementia post-diagnosis in England;
ii. Availability of all stakeholder groups involved in the Recovery College dementia course, namely staff, people with dementia, family/friend supporters involved in **a.** co-production and/or delivery and **b.** as course attendees

Sample

A purposive sample will include characteristics highlighted through our realist review as most relevant for developing the programme theory. We will sample across all stakeholders see figure 1, and across methods of delivery online and in person. Reflecting the diversity of the populations Recovery Colleges

serve and the types of courses they provide, will enable analyses to reflect and illuminate developments in different contexts. Based on our realist review these are:

Inclusion Criteria

- NHS Recovery Colleges which support people with dementia accessing memory services, including people from diverse ethnic and cultural local populations, socioeconomic and deprivation indices, urban/rural population mix;
- Recovery Colleges offering virtual or hybrid as well as traditional face-to-face courses;
- Courses offered by different staff groups i.e. psychologists, nurses, doctors, occupational therapists, support workers/assistant practitioners;
- Courses that involve both observable co-production and co-delivery of the course.

Exclusion criteria

- Courses provided for or predominantly attended by family carers or staff;
- Courses not co-produced with people with dementia.

A Recovery College dementia course co-production group typically ranges from 2 - 4 people; we plan to recruit and interview all who consent; across 5 case sites this will yield a sample of around n=15 individuals. These will include people living with dementia, family supporters and staff all involved in co-production and co-delivery of the course. Staff could be nurses, psychologists, support workers/assistant practitioners, doctors, occupational therapists, other allied healthcare professionals or service support staff. (see Figure 1 for example of stakeholder groups)

INSERT Figure 1 Stakeholders in Recovery College courses

The usual number of attendees at Recovery College dementia courses ranges from 8 - 20 people. Across 5 case sites this would yield a recruitment pool of between 40-100 people. We will purposively sample to recruit n=3-5 people per site ranging from people living with dementia, family/friend supporters and staff.

To examine service provision, we aim to recruit n=1-2 managers per site, who could be memory service managers/clinical team leaders or Recovery College leads/representative staff supporting Implementing Recovery for Organisational Change (ImROC) work in NHS mental health Trusts, giving a sample of 5-10 managers.

Consent

Once confirmation of capacity and capability has been received from each participating NHS mental health Trust and a Lead professional identified, those people co-producing the course will be consented into the study. Then as people enrol on the Recovery College dementia course, they will be advised of the planned research and invited to find out more about the study. PPI advisors have suggested the following words to use for the initial approach to each person booking a place on the course: *"Would you mind if two researchers join your course group?"*. At this point for people who do not wish to be involved in the research, the Lead Professional will coordinate their individual booking to alternative courses/dates. We acknowledge this may cause small inconvenience to people not wanting to be involved in the observation research, however this process was suggested by clinicians and PPI advisors and agreed by ethical review committee. See supplementary file 2 for further details on valid informed consent processes and consent flow chart.

For peer review only - http://bmjopen.bmj.com/site/about/guidelines.xhtml

Data collection

Within each case we will collect three types of data: **1** focused ethnography, **2** realist interviews and **3** documentary evidence. Data collection will take place over 18 months to provide time to capture data across one whole course.

1 Focused ethnography

Focused ethnography methods use short, intensive, linked field visits (22, 23). This method is pragmatically, ethically and methodologically appropriate here in not imposing an unduly heavy burden on people. In each case study site two of the three realist evaluation researchers (LB, JW, MH) will carry out ethnographic observations, potentially with a PPI advisory group member as a co-researcher. The co-researcher may make notes if they chose but they will have a reflective conversation with the researchers after each observation event. All will be situated as 'embedded participants' among course attendees. Each case study will entail a minimum of four hours observation but depending on length of preparation and course, this may be up to 14 hours. The findings derived from our realist review and subsequent PPI and staff advisory group discussions will further direct the focus for observations. A post-session debrief and reflection period held with the course co-producers/facilitators will explore whether having researchers as embedded participants changed the dynamics or behaviours in the room. Researchers will transcribe their handwritten field notes up as soon as possible after the observation, within 72 hours. Materials relevant to the observation such as floor plans or course materials will be stored alongside observations.

2 Realist interviews (24) After the course is delivered, we will conduct individual semi-structured qualitative interviews with course co-producers and attendees as well as NHS managers in memory services or Recovery College staff. Interviews will be undertaken within two weeks of the course finishing and may be face-to-face or virtual depending on the participants preference. They will be recorded and professionally transcribed verbatim. Interview topic guides were developed based on the findings of the realist review and together with the PPI advisory group. They include questions adapted to each stakeholder group; see supplementary file 3 for topic guides. Using focused ethnography initially will provide the researchers with detailed in-depth understanding of what happens in practice, and this will then inform interviewing to interrogate areas of tension, complexity and uncertainty. If participants become distressed or anxious during the interview the researcher will pause check their ongoing agreement to the interview and continue to finish the interview. The researcher will ensure the participant is orientated not distress and if required supported by a relative or friend before leaving. A second interview will be offered to course co-producers and attendees who wish to speak specifically about meeting the needs of ethnic and cultural minority groups in their courses. A brief summary of key points from the interview transcripts will be made available on request for participants to help people, particularly participants with dementia, to revisit their conversation, as was suggested by the PPI advisory group. This provides a chance for participants to change or add anything to the summary.

3 Documentary evidence We will review relevant documents such as course materials and evaluations to provide context and some triangulation with observations and interviews. For example, whether anonymised evaluations resonate with experiences discussed in interviews.

Data analysis

For peer review only - http://bmjopen.bmj.com/site/about/guidelines.xhtml

All relevant data will be uploaded into NVivo (25), a software package that supports qualitative data analysis. An iterative approach to analysis will enable emerging ideas to be refined and further explored in later case studies. We will analyse data collected using a realist logic (20). Data coding will be deductive (informed by our initial programme theory developed from the realist review), inductive (derived from the collected data) and retroductive (making inferences about mechanisms based on interpretations of our data to infer underlying causal processes). Questions on interpretations and judgement will guide the focus of analysis as outlined in **Table 1**.

Table 1. Questions to direct analysis of data from ethnography, interviews and documentary evidence

Relevance	Is source text section relevant to programme theory development?
Interpretation of meaning	If yes, does text content provide data that might be interpreted as functioning as **context, mechanism or outcome (C, M or O)**?
Interpretation and judgement about CMO configurations	For data that functions as C, M or O, which **CMO configuration (CMOC)** (partial or complete) does it belong to?<br>Can additional data informing this particular CMOC be found either in this source or other sources? If so which sources? How does this CMOC relate to other already-developed CMOCs?
Interpretations and judgements about programme theory	How does this particular (full or partial) CMOC relate to the programme theory?<br>What data from our sources inform how the CMOC relates to the programme theory? If yes, which sources provide which data?<br>Does the construction of this specific CMOC and any supporting data, require the programme theory to be changed?

Using a case study approach means that interpretations such as inferred mechanism of outcome can be examined and compared across cases to better explain why and how observed outcomes occur. We can compare 'successful' with 'less successful' cases of Recovery College courses, to examine how specific contexts or mechanisms have influenced outcomes observed. When using the questions in **Table 1**, we will also use the following forms of reasoning to make sense of the data:

- Juxtaposing: for example, where data about behaviour change in one source enables insights into data about outcomes in another source.
- Reconciling: where data differs between apparently similar circumstances (i.e. interviews and documentary review), it is appropriate to seek potential explanations for why these differences having occurred.
- Adjudicating: in terms of methodological strengths or weaknesses of the data sources.
- Consolidating data: where outcomes differ in particular contexts, constructing an explanation of how and why these outcomes occur differently.

Reporting of results will be guided by RAMESES (Realist And MEta-narrative Evidence Syntheses: Evolving Standards) (26). The findings will be used in another work package to co design resources and materials for providers of Recovery colleges in dementia.

In summary, the aim of this realist evaluation data collection and analysis is to specify theoretical claims that are grounded in data and that may be made transferable to real world contexts. Examining interview, observation and document data alongside each other will enable us to identify and apply differing perspectives and understandings, to refine the programme theory, in relation to experiences and discussions of dementia.

Data management

All confidential data such as consent forms and other study documentation will be archived securely for a period of 10 years in accordance with sponsor policy. Interview recordings will be transcribed and pseudonymised then destroyed. All data will have any identifying features removed. Data for analysis will be managed in NVivo (25). To support the development of the programme theory, all study data, such as interview transcripts, observations, notes from stakeholder and researcher analysis meetings are stored in a central file and ordered by date and type. Non-identifiable data sets will be made available to other researchers at reasonable request. Data management and storage will comply with UK General Data Protection Regulations (27).

Ethics and Dissemination

An important ethical concern in this study is gaining and monitoring consent from people living with dementia who may have fluctuating capacity. We will use the principles of process consent (28) - see supplementary file 2. Ethical approval was given by Coventry & Warwickshire Research Ethics Committee (22/WM/0215). This is a committee flagged for expertise in studies in dementia. A PPI advisory group member living with dementia attended the review meeting with the research team. The person found this an interesting experience and were active in directly addressing questions from the ethical review committee.

Learning from this research will be used in the following study work package where we will co-produce guidance and resources for groups planning or currently delivering Recovery College courses in dementia. Additional findings and learning will be disseminated through peer-reviewed publications, conferences, lay reports and via our website https://www.nsft.nhs.uk/discovery-study/.

Discussion

This ethnography draws on realist evaluation approaches to analyse, interpret and judge theoretical implications of data from naturally-occurring Recovery College courses in dementia. It is likely to be the first study to report rigorously analysed findings, using a realist approach, from what happens during the co-production and co-delivery of such courses to include the perspective and actions of people living with dementia, their family and friend supporters and healthcare staff, and how they affect each other in the context of each setting. This means identifying, recording and integrating outcomes and mechanisms for the course attendees and not only for the peer tutors and staff. This evaluation of Recovery College dementia courses will provide comprehensive evidence on their place within post-diagnostic support pathways in dementia, essential for implementing evidence-based practice in Recovery College dementia courses.

References

(1) Rewerska-Juśko M, Rejdak K. Social Stigma of People with Dementia. *J Alzheimers Dis*. 2020;78(4):1339–1343. doi: 10.3233/JAD-201004. PMID: 33185610.

(2) Herrmann LK, Welter E, Leverenz J et al. A Systematic Review of Dementia-related Stigma Research: Can We Move the Stigma Dial?. *Am. J. Geriatr. Psychiatry*. 2018;26(3):316–331.

For peer review only

(3) Aldridge H, Fisher P, Laidlaw K. Experiences of shame for people with dementia: An Interpretative Phenomenological Analysis. *Dementia*. 2019;18(5):1896–1911. doi: 10.1177/1471301217732430

(4) O'Shea E, Keogh F, Heneghan C. Post-Diagnostic Support for People with Dementia and their Carers (online). 2018. https://www.understandtogether.ie/news-and-events/news /Dementia-Post-diagnostic-Support-Literature-Review.pdf (Accessed 08 September 2020).

(5) Gauthier S, Webster C, Servaes S et al. 2022. World Alzheimer Report 2022: Life after diagnosis: Navigating treatment, care and support. London, England: Alzheimer's Disease International.

(6) Copland E, Hodge S, Clary L, Cartwright V. Memory Services National Accreditation Programme (MSNAP) Standards: Standards for Memory Services (online). 2018. https://www.rcpsych.ac.uk/docs /default-source/improving-care/ccqi/quality-networks/memory-clinics-msnap/msnap-standards-6th-edition-2018.pdf (Accessed 03 March 2021).

(7) Frost R, Walters K, Wilcock J et al. Mapping post-diagnostic dementia care in England: an e-survey. *J Integr Care*. 2020;29(1):22–36.

(8) Wheatley A, Bamford C, Brunskill G et al. Task-shifted approaches to post-diagnostic dementia support: a qualitative study exploring professional views and experiences. *BMJ Open*. 2020;10(9):1–8.

(9) Perkins R, Repper J, Rinaldi M, Brown H. Recovery Colleges. ImROC briefing 1 (online). 2012. https://imroc.org/wp-content/uploads/2022/04/1.Recovery-Colleges.pdf (Accessed 07 June 2023).

(10) Perkins R, Hill L, Daley S et al. 'Continuing to be me' – Recovering a life with a Diagnosis of Dementia. ImROC briefing 12 (online). 2016. https://imroc.org/resource/12-continuing-to-be-me-recovering-a-life-with-a-diagnosis-of-dementia/ (Accessed 07 June 2023).

(11) Perkins R, Meddings S, Williams S, Repper J. Recovery Colleges 10 Years On. ImROC briefing 15 (online). 2018. https://imroc.org/wp-content/uploads/2022/04/ImROC-Recovery-Colleges-10-Years-On.pdf (Accessed 07 June 2023).

(12) Leamy M, Bird V, Le Boutillier C et al. Conceptual framework for personal recovery in mental health: Systematic review and narrative synthesis. *Br J Psychiatry*. 2011;199(6):445–52.

(13) Department of Health. Living well with dementia: A National Dementia Strategy. 2009. Living Well With Dementia: a national dementia strategy - GOV.UK (www.gov.uk) (Accessed 01 March 2023).

(14) Hill L, Roberts G, Wildgoose J et al. Recovery and person-centred care in dementia: Common purpose, common practice? *Adv Psychiatr Treat*. 2010;16(4):288–98.

(15) Wolverson E, Hague L, West J, Teague B, Fox C, Birt L, Mills R, Rhodes T, Sams K, Moniz-Cook E. Building an understanding of UK Recovery College dementia courses: A national survey of Recovery College and memory services staff. *Work Older People*. 2023. In Press.

(16) O'Hagan M. Recovery Article for Mental Health Today. online 2009 http://www.maryohagan.com/resources/Text_Files/Recovery%20for%20Mental%20Health%20Today.pdf Accessed on 10.10.23

(17) Perkins, R. and Repper, J. (2021), "Editorial", *Mental Health and Social Inclusion*, Vol. 25 No. 1, pp. 1-6. https://doi.org/10.1108/MHSI-02-2021-082

(18) Perkins R, Repper J. Editorial Reclaiming Recovery. *Ment Heal Soc Incl*. 2021;25(1):1–6. doi: 10.1108/MHSI-02-2021-082

(19) Lowen C, Birt L, West J. Recovery colleges and dementia courses – a scoping survey. *Ment Heal Soc Incl.* 2019;23(4):166–72.

(20) Pawson R, Tilley N. Realistic Evaluation. London, SAGE Publications 1997.
(21) Yin RK. Case study research and applications: Design and methods. 6th ed. SAGE Publications 2017.
(22) Wall S. Focused Ethnography: A Methodological Adaptation for Social Research in Emerging Contexts. *Forum Qual Sozialforsch / Forum Qual Soc Res*. 2015;16(1):1–11.
(23) Knoblauch H. Focused Ethnography. *Forum Qual Sozialforsch / Forum Qual Soc Res.* 2005;6(3): 1-11.
(24) Manzano A. The craft of interviewing in realist evaluation. *Evaluation.* 2016;22(3):342–360. doi: 10.1177/1356389016638615
(25) QSR International Pty Ltd. (2020) NVivo (released in March 2020), https://www.qsrinternational.com/nvivo-qualitative-data-analysis-software/home
(26) Wong, G., Westhorp, G., Manzano, A. *et al. RAMESES II reporting standards for realist evaluations. BMC Med* ***14, 96 (2016). https://doi.org/10.1186/s12916-016-0643-1***
(27) Guide to the UK General Data Protection Regulation (UK GDPR) (online). 2018. https://ico.org.uk /for-organisations/guide-to-data-protection/guide-to-the-general-data-protection-regulation-gdpr/ (Accessed 08 June 2023).
(28) Dewing J. Participatory research: a method for process consent with persons who have dementia. *Dementia*. 2007;6(1):11–25.

For peer review only

Figure 1 Stakeholders in Recovery College courses

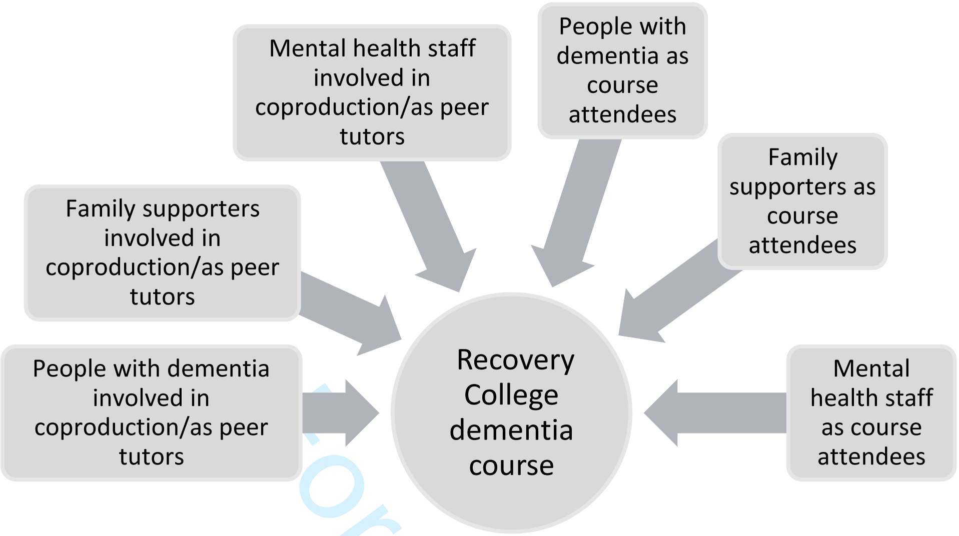

For peer review only

Supplementary file 1

Recovery Colleges

The first UK Recovery College was set up in 2009, growing to 85 by 2017 (Anfossi, 2017). The model has been replicated internationally (Perkins et al., 2018) with Recovery Colleges now in existence or developing in 22 countries (King & Meddings, 2019). Recovery College courses offer access to distinctive peer support both from co-producing courses and/or attending them (Sommer, Gill & Stein-Parbury, 2018). A national strategy to promote dementia peer support through developing learning networks also aims to empower people living with dementia to make choices in planning for the future (Department of Health, 2009). A 'recovery-focused', peer-support, adult learning approach is adopted and embedded within a strategic care delivery approach in mental health Trusts, to improve care outcomes beyond a narrow focus on symptom reduction, to help people rebuild meaningful, satisfying lives, despite limitations caused by mental health difficulties (Perkins et al., 2018). Recovery Colleges create adult learning environments which moderate power dynamics between service users and staff, to reduce stigma and increase attendees' sense of hope and empowerment (Meddings et al., 2014; Sommer, Gill & Stein-Parbury, 2018; Zabel et al., 2016). Attendees report developing novel coping strategies, improving self-worth, wellbeing and quality of life (Meddings et al., 2014; Rinaldi, Marland & Wybourn, 2011; Secker & Wilson, 2014; Zucchelli & Skinner, 2013).

A typical adult mental health Recovery College offers courses on mental health and recovery, designed to increase attendees' knowledge, skills and confidence in self-management of their own mental health and wellbeing. Of note, what 'wellbeing' means for people with dementia may be very different, but has been defined as emotional (positive states), social (connections and belonging) and psychological (positive sense of self, going beyond and agency and purpose) with an overarching theme of 'valuing life', which differs to studies evaluating life satisfaction (Clarke et al., 2020). Courses range from one-off sessions to several sessions spread over a set number of weeks. All courses are co-produced and co-delivered (i.e. co-taught) by peer tutors - that is, people with lived/expert experience - and mental health staff, who also enrol on courses as attendees. Peer tutors prepare for their role through having training to teach and support, and receive supervision to ensure any sensitive issues can be supported effectively.
A theory of change model for Recovery Colleges has been co-developed within adult mental health contexts that identifies four mechanisms of change (Toney et al., 2018) empowering environment - opportunities for choices; shifting balance of power; enabling different relationships and connecting with peers; and facilitating personal growth through shared learning and strength-building. Outcomes were changes in the attendee including improved wellbeing, reinforced by life changes they could observe. This model is highly applicable to enabling desired outcomes for post-diagnostic support in dementia.

A 'recovery-focused', peer-support, adult learning approach is adopted and embedded within a strategic care delivery approach in mental health Trusts, to improve care outcomes beyond a narrow focus on symptom reduction, to help people rebuild meaningful, satisfying lives, despite limitations caused by mental health difficulties (Perkins et al., 2018). This approach is already flourishing in adult mental health services, encouraged since 2011 by the Department of Health commissioned 'Implementing Recovery through Organisational Change' (ImROC) collaborative https://imroc.org/. Working with mental health Trusts, ImROC founded Recovery Colleges which have rapidly become a core part of recovery-focused mental health services. As of 2017, there were 85 Recovery Colleges in the UK (77 in England, 2 in Scotland, 5 in Northern Ireland and 1 in Wales; Anfossi, 2017).

For peer review only - http://bmjopen.bmj.com/site/about/guidelines.xhtml

Recovery Colleges further have five key linked conceptual processes - the **CHIME** recovery framework – robustly developed to underpin the term 'recovery' in this context: **connecting** with others, inspiring **hope**, maintaining a positive **identity**, finding **meaning** in life outside of symptoms and **empowering** control over life and a focus on strengths (Leamy et al., 2011).

The CHIME Recovery framework, as operationalised through Recovery Colleges, has clearly relevant links with the NICE-recommended person-centred care framework for dementia (Leamy et al., 2011; Brooker, 2007), the Royal College of Psychiatrists Memory Services National Accreditation Programme (MSNAP; Copland et al., 2018) and the National Dementia Strategy objective to develop peer support and learning networks (Department of Health, 2009). Key domains for person-centred care are **valuing** people living with dementia and those (both informal family and friends and health and social care staff) who care for them; providing care that is **individualised**; understanding and acting from the **perspectives** of people living with dementia (which can reinforce connections, meanings and identities); and creating positive **social-psychological** environments (which can build hope and empowerment; Brooker, 2007). For dementia care to be person-centred, all these elements are needed and need to work together. Both frameworks require mental health services to continuously improve, and to evidence progress towards more meaningful care within each of these domains (Brooker, 2007; Leamy ey al., 2011,) yet person-centred principles are still poorly and inconsistently translated into practice. Applying these frameworks in memory post-diagnostic support services is important for keeping a whole person focus so as to value the identity, perspectives, strengths and needs of people living with dementia, and so to enable staff to share with patients more effectively care planning alongside understandings of personal recovery in adjusting to dementia.

Five recovery processes giving the acronym CHIME

**Connectedness** with others;<br>inspiring **Hope** and optimism about the future;<br>maintaining a positive **Identity**;<br>finding **Meaning** in life outside of symptoms;<br>and **Empowerment** with control over life and a focus on strengths. (Leamy et al., 2011)

References

Anfossi A. The current state of Recovery Colleges in the UK : final report. *ImROC.* 2017.

Brooker D. *Person-centred Dementia Care: Making services better.* London: Jessica Kingsley; 2007.

Clarke C, Woods B, Moniz-Cook E, Mountain G, Øksnebjerg L, Chattat R, Diaz A, Gove D, Vernooij-Dassen M, Wolverson E. Measuring the well-being of people with dementia: a conceptual scoping review. *Health and quality of life outcomes.* 2020;18(1), 1–14

Copland E, Hodge S, Clary L, Cartwright V. *Memory Services National Accreditation Programme (MSNAP) Standards: Standards for Memory Services*. London; 2018. Available from: https://www.rcpsych.ac.uk/docs /default-source/improving-care/ccqi/quality-networks/memory-clinics-msnap/msnap-standards-6th-edition-2018.pdf [Accessed 3rd March 2021].

Department of Health. *Living well with dementia: A National Dementia Strategy.* 2009. Available from: https://assets.publishing.service.gov.uk/government/uploads/system/uploads/attachment_data/file /168220/dh_094051.pdf [Accessed 10th December 2019].

King T, Meddings S. Survey identifying commonality across international Recovery Colleges. *Ment Heal Soc Incl.* 2019;23(3):121–8.

Leamy M, Bird V, Le Boutillier C, Williams J, Slade M. Conceptual framework for personal recovery in mental health: Systematic review and narrative synthesis. *Br J Psychiatry*. 2011;199(6):445–52.
Meddings S, Byrne D, Barnicoat S, Campbell E, Locks L. Co-Delivered and Co-Produced: Creating a Recovery College in Partnership. Jce . Vol 9 (1); pp16-25. *J Ment Heal Training, Educ Pract.* 2014;9(1):16–25.
Meddings S, McGregor J, Roeg W, Shepherd G. Recovery Colleges: Quality and outcomes. *Ment Heal Soc Incl*. 2015;19(4):212–21.
Perkins R, Meddings S, Williams S, Repper J. Recovery Colleges 10 Years On. *ImROC.* 2018
Rinaldi M, Marland M, Wybourn S. *Annual Report 2011 – 2012 South West London Recovery College*. 2012. Available from: http://rfact.org.au/wp-content/uploads/2015/05/SW-London-Recovery-College-evaluation-2011_12-v1-0.pdf [Accessed 12th February 2020].
Secker J, Wilson C. *Evaluation of the mid Essex Recovery College, October – December 2013. 2014.* Anglia Ruskin University, 2014.
Sommer J, Gill K, Stein-Parbury J. Walking side-by-side: Recovery Colleges revolutionising mental health care. *Ment Heal Soc Incl*. 2018;22(1):18–26.
Toney R, Elton D, Munday E, Hamill K, Crowther A, Meddings S, et al. Mechanisms of action and outcomes for students in recovery colleges. *Psychiatr Serv.* 2018;69(12):1222–9.
Zabel E, Donegan G, Lawrence K, French P. Exploring the impact of the recovery academy: a qualitative study of Recovery College experiences. *J Ment Heal Training, Educ Pract*. 2016;11(3):162–71.
Zucchelli FA, Skinner S. Central and North West London NHS Foundation Trust's (CNWL) Recovery College: the story so far. *Ment Heal Soc Incl*. 2013;25

For peer review only

Supplementary file 2 Valid Informed Consent Processes

Process of obtaining consent from each co-production group The co-production group at each site will be contacted first and we aim to be as flexible and pragmatic as possible in recruiting and seeking informed consent from individuals in the group so we can begin observing co-production activities and documents as soon as possible prior to the researcher-observed course starting.

- Two case study researchers arrange an informal pre-course meeting with the co-production group to seek permission to contact for individual consent meeting/s
- Facilitate introductions, give information about the study, answer any questions, discuss and plan to join further meetings as ethnographic observers of the co-production process
- Gain understanding of practicalities around course publicity, set up and co-facilitation
- Discuss what types of documents and materials might be available for researchers to review

Process of obtaining consent from attendees Similarly we aim to be as flexible and pragmatic as possible in recruiting and seeking informed consent from all dementia course attendees. When people enrol on the Recovery College dementia course, they will be advised of the planned research and invited to find out more about the study. If at this point people do not wish to be involved in the research, the Lead Professional will coordinate their individual booking to alternative courses/dates.

If a person agrees in principle, the first line of obtaining consent will be for attendees who inform the case site Lead Professional they are planning to attend the course. The Lead Professional/site staff will provide the attendee with a participant information sheet. If agreed with the potential attendee, a 'permission to contact' form will be completed by the Lead Professional/site staff and sent securely via email to the study team via the DiSCOVERY study email address. The study team will attempt to obtain informed consent from all attendees, including healthcare professionals, people with dementia and their family supporters via remote methods prior to the day of data collection. If remote methods of informed consent are not possible, attendees will be offered the opportunity to speak to the study team in person prior to the course and provide informed consent on the day. However it is possible that people will attend the course without providing prior consent for taking part in the study. In those cases, attendees will be provided study information and offered the opportunity to take part in the research study immediately prior to the data collection commencing in accordance with HRA guidance for applying a proportionate approach to the process of seeking consent (Health Research Authority, 2017) and researchers will be mindful and adapt their approach by slowing the process down to enable people with dementia seeking post-diagnostic support via a Recovery College dementia course to make an informed decision.

Once dates for the research course are identified and agreed, researchers will plan to attend and observe sessions. All in the room will be reassured that researchers are not looking at individuals' 'performance', but rather looking at how the course is experienced by everyone in the room. In the interests of data transparency and inclusion, everyone involved will be given the opportunity to view researchers ethnographic record sheets if people so wish.

Following the course, researchers will invite co-producers and attendees to take part in individual one-to-one interviews and/or focus groups which will be arranged individually by telephone and/or email following the end of the course.

Supporting information for the initial process for obtaining valid informed consent We will provide each site with Participant Information Sheets before each stage of consent and data collection. Participant Information Sheets will make clear that participation is voluntary and that participants will have opportunities to discuss the study, ask questions and ask for further information before we ask individuals to give their informed consent. We will use a variety of methods for recording consent, according to the needs and wishes of each individual, either via post and telephone, or using Microsoft Teams/Zoom to record the completion of the consent form. Verbal consent will also be confirmed on each occasion we meet people.

Supporting people experiencing fluctuating mental capacity to participate if they wish Most people diagnosed in NHS memory services are likely to have mild to moderate dementia symptoms in the post-diagnostic phase. We are aware that for people living with a progressive cognitive impairment, fluctuating mental capacity can affect a person day-to-day, hour-to-hour and moment-to-moment, and that these experiences are different for each person. People with dementia may also experience difficulties communicating (both verbal and written) and responding to questions due to cognitive and/or sensory impairments. Participants who may lack capacity to consent will not be excluded, and consent will be treated as an ongoing process reviewed at each point of contact. Researchers will use a proforma for recording capacity assessment for people with dementia to give consent developed by the study Sponsor research department. In the event that a potential participant has fluctuating capacity, they will be asked what they would like to do if their capacity were to deteriorate, and if they have a trusted person we could speak to. Where people lack capacity, we will seek advice from a personal consultee (usually a person's family/friend supporter) on what the wishes and feelings of the person might be, and whether or not they should take part.

Process consent for ethnography in a group setting The case study site researchers responsible for obtaining valid informed consent, have extensive experience of delivering NIHR portfolio dementia studies using mixed methods in health and social care settings and will be observing the principles of process consent (Dewing, 2007) in dementia research and data collection at case study sites. They have extensive experience of using observational methods in research and clinical dementia care practice settings. Closely observing individuals at all stages of dementia for verbal and non-verbal signs of well and ill-being is central to interviewing, ethnographic and analysis activities, to address supporting participation when and where appropriate, and in a person-centred, inclusive and enabling way, to ensure all individuals viewpoints are heard. All stakeholders in the selected course will have been invited to participate and valid informed consent will have been established prior to the course start as detailed above. Adopting a process consent approach (Dewing, 2007) will guide practice during the observations by watching for any signs which may indicate a non-verbal withdrawal of consent by individuals. If this is the case, we won't stop the observations, but we will not record any further actions from the individual.

Ensuring information is accessible We will be flexible in the ways in which we communicate on the day/s of ethnography and individual interviews, for example providing information at different times, allowing extra time, introducing ourselves and drawing on our person-centred interpersonal skills. The PPI advisory group have shared with us some challenges of experiencing short-term memory difficulties in, for example, feeling less able to concentrate for usual-length meetings via Zoom. The group have helped improve accessibility of participant information sheets and recruitment materials. Where possible, we will also identify people who may have English language and/or reading and writing needs, and will engage

interpreters/translators where required, alongside the use of visual aids. For people who arrive on the day of the course session without prior informed consent, we will pragmatically ask family supporters if they are comfortable to act as a personal interpreter if needed. The research team are experienced in working with people with language needs, and neurological impairments, and have successfully used these methods in prior research studies to support inclusion and respect.

Process for recording consent the PPI advisory group suggest offering a range of choices to support this process after receiving and talking through the Participant Information sheet:

- On paper receiving a consent form by post and completing and returning via a pre-paid return envelope (using the Sponsor organisation FREEPOST service);
- On paper with a researcher face-to-face;
- Electronically via a secure Microsoft Forms link sent by email;
- Electronically by recording the consent part of the recruitment conversation online with a researcher via video platform.
- In person with local Clinical Research Network nurses

References

Health Research Authority. *Applying a proportionate approach to the process of seeking consent.* HRA Guidance. 2017. Available from: https://s3.eu-west-2.amazonaws.com/www.hra.nhs.uk/media/documents /applying-proportionate-approach-process-seeking-consent_R3gbJKn.pdf [Accessed 02nd August 2022].

Dewing J. Participatory research: a method for process consent with persons who have dementia. *Dementia*. 2007;6(1):11–25.

For peer review only

Flowchart illustrating points of consent activity:

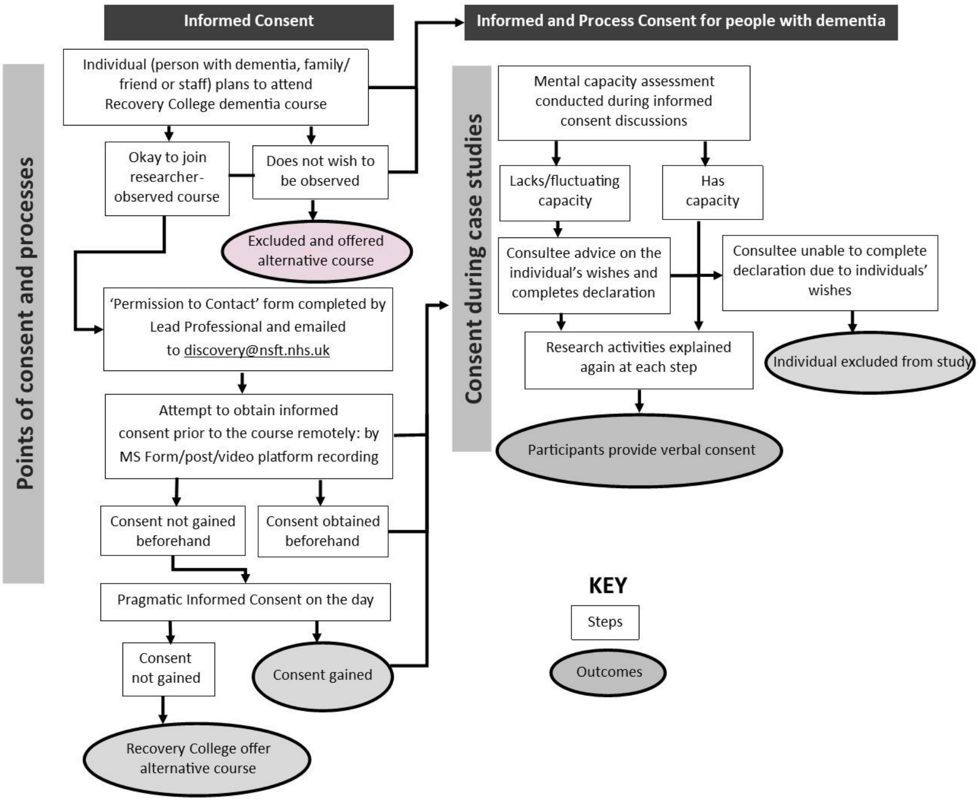

For peer review only - http://bmjopen.bmj.com/site/about/guidelines.xhtml

Topic guide - coproducers/facilitators - people with dementia and supporters

Can you tell me about your experiences of co-producing and facilitating a Recovery College dementia course?

How did you become involved? Why did you become involved?

- *approached by a member of staff*
- *through the central RC*
- *word of mouth or advert*

What have been the benefits of being involved?

What have been the challenges of being involved?

Can you tell me more about your role in co-developing the course?

- *who were you working with?*
- *how often did you meet and where*?

Was there already an idea for the course and how much do you think you helped to develop the course?

Were you happy with the level of involvement you had with developing the course?

Can you expand on that?

What support was provided for you as a coproduction partner?

- *financial*
- *practical (with technology/travel/accessing venues)*
- *emotional*

Who provided this support?

For peer review only - http://bmjopen.bmj.com/site/about/guidelines.xhtml

Did you experience any challenges for co-developing the course?

- *practical*
- *emotional*
- *time commitments*

How was your experience of working with the staff to co-produce the course?

- *supportive*
- *different from usual encounters with staff*
- *differences in power relations*

What do you see you gained from being involved with developing the course?

- *e.g. sense of purpose, new skills, confidence in skills, contributing by sharing own experience with others*

From your experience, do you think there are certain skills or motives that have helped you to co-produce the course?

- *confident with sharing experience*
- *willing to contribute*
- *ready to share experiences – can you expand on this?*

Can you tell me about your experience of running the course?

What support do you think you needed to run the course?

- *what worked well*
- *what did not work so well*

What do you think you have gained from running the course?

- *increase confidence*
- *chance to give back*

What do you think people gain from you running the course?

- *ideas that might help people live positively*
- *learning from and sharing experiences with others*
- *seeing someone with dementia leading the course and changing attitudes towards dementia*

For peer review only - http://bmjopen.bmj.com/site/about/guidelines.xhtml

- *increased knowledge of resources outside of the course (this could include links with other people)*

Are there negatives for you in running the course?

- *time-consuming*
- *difficult to talk about experiences?*
- *tiring*
- *level of expected involvement may not be in line with actual involvement*

Where and how is the course run?

- *online*
- *face-to-face*
- *what kind of venue?*
- *how often?*
- *for how long?*

Which do you prefer?

- *How do you think this impacts your experience of running the course?*
- *Why*

Which groups of people have you noticed come to the course?

- *people with dementia/supporters/staff?*
- *particular age group?*
- *background/ethnicity?*

Are there particular people/groups that you think aren't attending? Who? Why do you think this is?

- *transport/unable to access online*
- *lack of confidence*
- *aren't aware of the Recovery College*
- *think 'recovery' isn't relevant for people with dementia*
- *do not relate to the people running or attending course so think it is not for them*

How could more people be encouraged to attend dementia courses?

- *changes to how course is advertised (e.g. language/more widely advertised)*
- *buddy system/support with travel/technology*)

For peer review only - http://bmjopen.bmj.com/site/about/guidelines.xhtml

Are you involved with evaluating the course?

- *How is the course evaluated? (standard feedback form/questionnaire, more open-ended feedback welcomed?)*
- *Is this information used to make changes to future courses?*
- *Does it influence whether the course is run again?*

Anything else you would like to add?

For peer review only - http://bmjopen.bmj.com/site/about/guidelines.xhtml

Topic guide - attendees - people with dementia and supporters

Can you tell me about your experiences of attending a Recovery College dementia course?

How did you hear about the course?

- *from a member of staff / care team*
- *word of mouth*
- *social media*

Why did you decide to attend?

- *wanted to meet others in a similar situation*
- *wanted to learn strategies to live positively with dementia*
- *wanted to find out what a Recovery College is*

Where and how was the course run?

- *online/face-to-face?*
- *what kind of venue?*

Did you have any worries or concerns before joining the course?

- *getting there / joining online*
- *didn't feel confident enough, worried about talking or meeting others,*
- *what you might be expected to do during the course*
- *how you might feel during or after the course*
- *not sure if recovery is relevant to someone with dementia*

Do you think things could have been done to make the course easier to access?

- If yes, in what way?

How could more people be encouraged to attend Recovery College dementia courses?

- *easier to understand language*
- *more widely advertised*
- *easier ways to sign up*
- *buddy system / support with travel / technology*

For peer review only - http://bmjopen.bmj.com/site/about/guidelines.xhtml

Would you prefer courses online or in person?

- Why?

What have been the positive outcomes from attending the course?

- *learning from and sharing experiences with others,*
- *seeing people with dementia running the course*
- *improving how I see myself and my situation*
- *knowing where I might get help or thinking about how I can better manage*

What have been the negative points from attending the course?

How do you think the course could have been improved?

- *ratio between people with dementia / family supporters and staff*
- *did everyone get the chance to speak if they wanted to?*
- *did you see it as a safe space?*

Anything else you would like to add?

For peer review only - http://bmjopen.bmj.com/site/about/guidelines.xhtml

Topic guide for staff coproducers/facilitators

General Qs

Can you tell me about your experiences of coproducing and facilitating a Recovery College dementia course?
How did you become involved? Why did you become involved?
What have been the benefits of being involved?
What have been the challenges?

Setting up a course

What factors impact the decision to set up a Recovery College dementia course?

- *Resources?*
- *Backing of certain individuals?*
- *Attitudes towards recovery in relation to dementia?*
- *Culture within the Trust?*

What is the relationship between memory services and the Recovery College?

- *How do they complement one another?*
- *Do they have differing aims?*
- *Do memory services signpost people to the Recovery College?*

Who is the course open to?

- *People who have accessed services, supporters?*
- *Staff?*
- *General public?*

How is the course advertised?

- *On social media?*
- *Through the Recovery College?*
- *Through services?*
- *Word of mouth?*
- *Any other*

Are there particular groups of people you are aiming to reach with the Recovery College courses and what methods do you use to reach them?
Does this work?

Coproduction

How important is coproduction to the course?

- Why is it important?

What have been your experiences of coproducing the dementia course?

What would co-production of the course ideally look like?

- Is it possible? Why, or why not?
    - Resources co-producers can access
    - Time
    - Decision making
- What level of co-production do you consider good enough?

For peer review only - http://bmjopen.bmj.com/site/about/guidelines.xhtml

What have been the benefits for you in coproducing the course?

- *Ability to work in line with values?*
- *Help to re-engage with day-to-day job*
- *Professional growth*
- *Personally rewarding (how?)*
- *Decreased stress/burnout?*

What are the challenges of coproduction?

- Embracing a different power dynamic/valuing each person's involvement? *(for you? For the people you're coproducing with?)*
- Amount of time it takes?
- Any difficulties with working with people with dementia specifically? (*If yes, what are they, how do you address and overcome these?*)

How do you find people to coproduce courses with?

- *People you've worked with clinically?*
- *Through the central Recovery College?*
- *Word of mouth?*

Are there certain skills, characteristics, or experience that you look for in a coproduction partner?

- *Confident with sharing their experience*
- *Willingness to contribute*
- *Emotionally 'robust' enough?*

Can you tell me about a time when you were struggling to work in a co-productive way and what you did to address this?

- *The fact that they might benefit from the process?*

What support is provided for coproduction partners (people with dementia and supporters)?

- Financial?
- Practical *(with technology/travel/accessing venues)?*
- Psychological/emotional?

Who provides this support?

Has coproducing the course impacted your day-to-day job?

- If yes, how and how have you managed this? *(Affected interactions with other people with dementia? More likely to apply recovery-focused principles?)*

Running a course

Where/how is the course run?

- *Online/face-to-face?*
- *What kind of venue?*

Do you think this impacts the delivery of the course?

- *Are people with dementia less able to use technology to attend online?*
- *Does the course being face-to-face encourage more discussion or building of relationships?*

What have been the positives of running the course?

For peer review only - http://bmjopen.bmj.com/site/about/guidelines.xhtml

- For you as a member of staff *(chance to gain holistic view of people with dementia, to see them recovering and thriving, increase personal wellbeing, changed attitudes towards recovery)*
- For peer tutors *(increase confidence, chance to give back)*
- For students-attendees (*empowerment and self-management, a safe space, learning from and sharing experiences with others, seeing peers leading and succeeding, reduced stigma and more positive sense of self, able to visualise living well, see self as more than diagnosis, ability to build relationships that last outside of the course)*

And the negatives? *(time-consuming alongside other responsibilities)*

Have you felt supported whilst running the course?
- *By the Trust (if applicable) at organisation lead level or manager level?*
- *By fellow coproducers?*
- *By other colleagues?*
- *With administrative staff?*

Who comes along to the courses?
- *People with dementia/supporters/healthcare staff?*
- *Particular age group?*
- *Background/ethnicity?*

Are there particular people that you think aren't attending?
- Why do you think this is?
  - *Transport/unable to access online*
  - *Lack of confidence*
  - *Aren't aware of the Recovery College*
  - *Think 'recovery' isn't relevant for people with dementia*
  - *Don't think the course is for them (why might that be?)*

How could more people be encouraged to attend dementia courses?
- *More accessible language*
- *More widely advertised*
- *Buddy system/support with travel/technology*
- *Ensuring adverts and materials 'speak' to the people trying to reach*

Evaluating a course
Do you evaluate the course?
- How? *(feedback form/questionnaire, more open-ended feedback welcomed?)*
- Is this information used to make changes to future courses?
- Does it influence whether the course is run again?

Anything else you would like to add?

For peer review only - http://bmjopen.bmj.com/site/about/guidelines.xhtml

Topic guide for attendees (staff)

Can you tell me about your experiences of attending a Recovery College dementia course?

How did you hear about the course?

- *From another member of staff*
- *Word of mouth*
- *Social media*
- *Through central Recovery College*
- *Previous attendance at Recovery College*

Why did you decide to attend?

- *Thought learning would be helpful in day-to-day role*
- *Wanted to learn more about recovery and dementia*
- *Wanted a different way of working with people with dementia*
- *Wanted to find out more about the Recovery College*

Were there any barriers to you attending?

- *Taking time out of work*
- *Permission from line manager*

Or how were you encouraged to attend and why?

- *Recommended by colleagues*
- *Part of CPD*
- *Considering role of coproducer*

Where and how was the course run?

- *Online*
- *Face-to-face? What kind of venue?*

Did this work for you? Why or why not?

Do you think the course could have been more accessible?

- If yes, in what way?

How could more people be encouraged to attend Recovery College dementia courses?

- *More accessible language (less recovery-focused?)*
- *More widely advertised*
- *Support with travel/technology*

What have you gained from attending the course?

- *Learning from and sharing experiences with others*
- *Seeing people with dementia leading, succeeding, and living positively*
- *Reduced stigma and changed attitudes towards people with dementia*

Were your expectations for the course met?

- *What were they?*
- *How and why or why not?*

For peer review only - http://bmjopen.bmj.com/site/about/guidelines.xhtml

How do you think the course could have been improved?

- *Ratio between lived and professional experience*
- *How the course was facilitated*
    - *Did everyone get the chance to speak if they wanted to*
    - *Did it feel like a safe space?*

What was your understanding of recovery in the context of dementia before attending? Did this change after attending the course?

- *Not recognised as important by colleagues*

Was there any learning that you will take back to your day-to-day role?

Anything you would like to add?

For peer review only - http://bmjopen.bmj.com/site/about/guidelines.xhtml
